# Constitutional isomerism of the linkages in donor–acceptor covalent organic frameworks and its impact on photocatalysis

**DOI:** 10.1038/s41467-022-33875-9

**Published:** 2022-10-23

**Authors:** Jin Yang, Samrat Ghosh, Jérôme Roeser, Amitava Acharjya, Christopher Penschke, Yusuke Tsutsui, Jabor Rabeah, Tianyi Wang, Simon Yves Djoko Tameu, Meng-Yang Ye, Julia Grüneberg, Shuang Li, Changxia Li, Reinhard Schomäcker, Roel Van De Krol, Shu Seki, Peter Saalfrank, Arne Thomas

**Affiliations:** 1 Department of Chemistry/Functional Materials, Technische Universität Berlin, Berlin, Germany; 2 Inorganic and Physical Chemistry Laboratory, CSIR─Central Leather Research Institute, Chennai, 600020 India; 3 Institute of Chemistry/Theoretical Chemistry, University of Potsdam, Potsdam, Germany; 4 Department of Molecular Engineering, Kyoto University, Kyoto, Japan; 5 Leibniz-Instituts für Katalyse e.V. an der Universität Rostock, Rostock, Germany; 6 Institute for Solar Fuels, Helmholtz-Zentrum Berlin für Materialien und Energie GmbH, Berlin, Germany; 7 Department of Chemistry/Chemical Reaction Engineering, Technische Universität Berlin, Berlin, Germany

**Keywords:** Metal-organic frameworks, Photocatalysis

## Abstract

When new covalent organic frameworks (COFs) are designed, the main efforts are typically focused on selecting specific building blocks with certain geometries and properties to control the structure and function of the final COFs. The nature of the linkage (imine, boroxine, vinyl, etc.) between these building blocks naturally also defines their properties. However, besides the linkage type, the orientation, *i.e*., the constitutional isomerism of these linkages, has rarely been considered so far as an essential aspect. In this work, three pairs of constitutionally isomeric imine-linked donor-acceptor (D-A) COFs are synthesized, which are different in the orientation of the imine bonds (D-C=N-A (DCNA) and D-N=C-A (DNCA)). The constitutional isomers show substantial differences in their photophysical properties and consequently in their photocatalytic performance. Indeed, all DCNA COFs show enhanced photocatalytic H_2_ evolution performance than the corresponding DNCA COFs. Besides the imine COFs shown here, it can be concluded that the proposed concept of constitutional isomerism of linkages in COFs is quite universal and should be considered when designing and tuning the properties of COFs.

## Abstract (web summary)

Systematic investigation of isomerism in covalent organic frameworks (COFs) can provide key insights into their properties. Here, the authors reveal that the constitutional isomerism of the linkage i.e., linkage orientations distinctly impact COFs’ structural and photophysical properties.

## Introduction

**Fig. 1:**
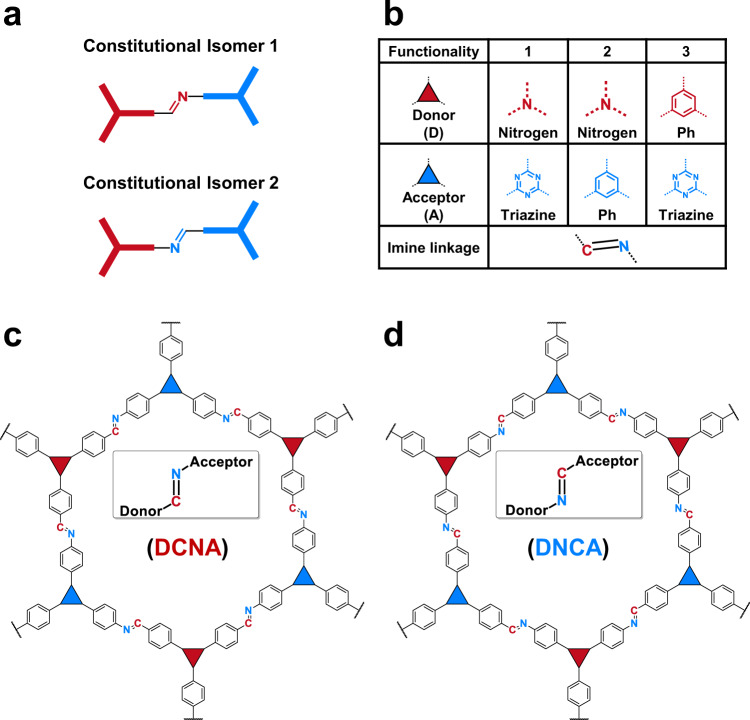
Chemical structures of the isomeric COFs. **a** Constitutional isomerism of the imine linkage. The red and blue triangles represent donor or acceptor moieties. **b** Building blocks for the COFs synthesis in this work. The chemical backbones of the isomeric COFs (**c**) DCNA and (**d**) DNCA in this work.

Covalent organic frameworks (COFs) are porous crystalline materials composed of organic building units (linkers) connected through strong covalent bonds^1–5^. Functionality and structural diversity are mostly introduced into such COFs by applying different linkers so far^6–9^. While there are intensive studies on linker chemistry, the COF linkages. i.e., the bonds formed between the linkers during crystallization, have gained much less attention even though they play a critical role in both for the design and the properties of COFs^10–12^. Currently, research on COF linkages is focused on exploring new linkages to increase the amount of available synthetic methods for reticulating organic building blocks but also for tuning the properties of the resulting COFs^9,13–17^. Strategies for post-synthetic modification of existing linkages, such as protonation^18,19^ or transformation to more stable covalent bonds^20–23^ were developed for the same purpose. Furthermore, synthetic methodologies to introduce mixed linkages such as boronate/boroxine and imine linkages into COFs have been described^24,25^. However, another obvious structural variation of currently available linkages, namely their orientation towards the linkers, has so far not been exploited in depth. Imine-linked COFs have been reported frequently due to the ease of their synthesis and relatively high chemical stability^10^. Such C=N-linked COFs are prepared by condensation of aldehyde- and amino-functionalized monomers, while there seems to be no rationale behind the choice, of which of the monomers should be aldehyde or amino-functionalized, respectively. Thus, presumably, just the cheaper or easier-to-prepare monomers are commonly chosen. However, this decision might significantly impact the properties of the final materials, as it predetermines the orientation of the C=N-linkage towards the different linkers yielding the possibility of constitutional (also called structural) isomerism within this connection (Fig. 1a)^26–28^. Some early studies described the different properties of the respective monomers when the amine and aldehyde functionalities are reversed, which yielded different growth kinetics and structural evolutions when forming single layer, surface-grown constitutional isomeric COFs^26,27^. In a very recent study, a pair of imine-linked constitutional isomers of COFs was compared, proving different reversibility between two crystal phases in which the COFs can exist^29^. In this work, also the different optical properties of the isomeric COFs are mentioned seen by a changed absorption and emission spectrum. Thus, current examples of constitutional isomeric COFs have mainly focused on synthetic aspects and structural properties, while a systematic study revealing the impact of constitutional isomerism of various imine-linked COFs has so far not been attempted.

The constitutional isomerism of the linkages might be especially important for donor–acceptor (D-A) type COFs, currently vastly explored for different optoelectronic or photocatalytic applications^8^, in which the interaction between the D and A moieties are crucial for the final properties and performance^30^. Indeed, Ghosh et al. observed at first no significant difference in the optical properties of two amorphous microporous polymer networks in which anthracene and pyrene moieties (thus two donors) were connected by imine bonds in reverse orientation. However, after doping with iodine, thus oxidation of the pyrene moiety, the crucial influence of imine linkage orientation on the electronic properties of the networks was revealed^31^.

In imine-linked D-A COFs, two isomeric structures can be imagined, one in which the donor is linked to the imine carbon (D-C=N-A) the other where it is linked to the imine nitrogen (D–N=C–A) (Fig. 1c, d). In this work, three D-A COFs and their constitutional isomers were prepared (Fig. 1) to investigate and compare their structural and optoelectronic properties as well as their photocatalytic performance. In the three pairs of isomeric D-A COFs, functional units with different electron donor and acceptor strengths are connected, allowing a comprehensive investigation on how the electronic coupling of such building blocks is influenced by the orientation of their linkage. We have recently shown that protonation of imine bond in COFs significantly change their optoelectronic properties and is a crucial preliminary step to activate such COFs as photocatalysts for hydrogen evolution from water^18^. In this work, we show that this is also valid for the pairs of constitutionally isomeric COFs.

## Results

### Synthesis and characterization of the isomeric COFs

To achieve different D-A combinations, triphenylamine, triphenylbenzene, and triphenyltriazine monomers were combined (Fig. 1b). The triphenylamine motif is a strong electron donor^32^ whereas, triazine is an electron-accepting building block^17^. Finally, the triphenylbenzene monomers can serve as both donor and acceptor depending on the counter building blocks^18^. The three pairs of isomeric COFs were named DCNA-*X* and DNCA-*X* (*X* = 1–3), in which D stands for donor, A for acceptor, while CN or NC shows how the imine linkage is connected to the D and A nodes (Fig. 1b). DCNA-1 and DNCA-1 thus combine the strongest donor and acceptor units, and it will be shown that the difference in their connectivity, i.e., the orientation of the imine linkage, has the most distinct effect on the respective properties of this pair of isomeric COFs. Therefore, mainly the results for these constitutional isomers are presented in the following. Comparative measurements of the other two isomeric COFs pairs, underlying the trend, are shown in the supporting information.

**Fig. 2:**
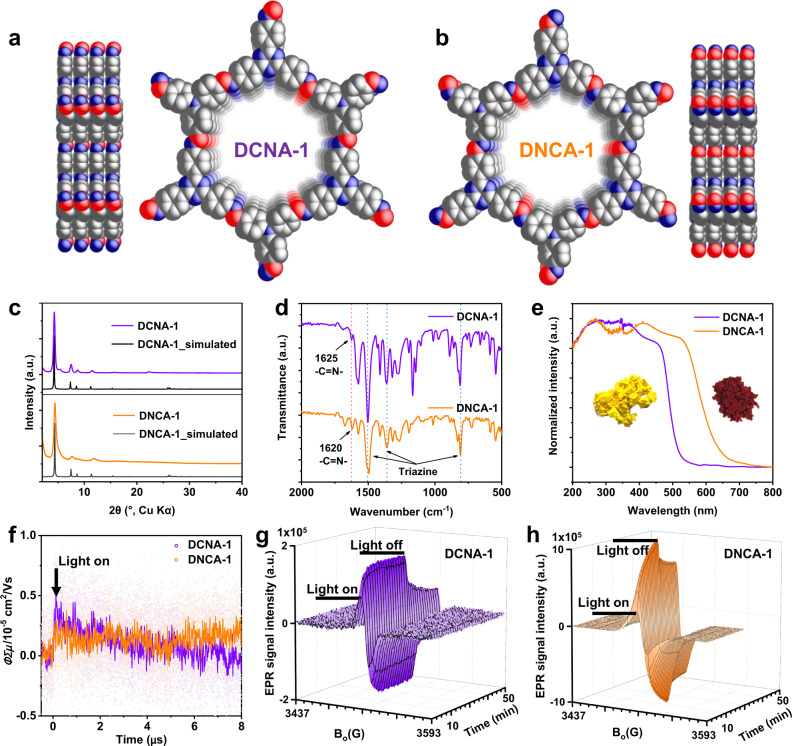
Characterization of the constitutional isomers DCNA-1 / DNCA-1. Structure models of the isomeric COFs DCNA-1 (**a**) and DNCA-1 (**b**) from the side and top views. (Red: imine carbon, Blue: nitrogen, Gray: phenyl or triazine carbon). **c** Experimental and simulated PXRD patterns, (**d**) FTIR spectra, (**e**) UV-Vis DRS spectra, and (**f**) flash-photolysis time-resolved microwave conductivity (FP-TRMC) transients of the isomeric COFs DCNA-1 and DNCA-1. Electron paramagnetic resonance (EPR) conduction band (CB) electrons spectra of DCNA-1 (**g**) and DNCA-1 (**h**) during and after visible light irradiation (>420 nm, 300 W Xe lamp). Insets in (**e**) show the corresponding photographs of DCNA-1 (yellow) and DNCA-1 (dark red).

Constitutional isomers of the COFs can be synthesized from the respective amine and aldehyde functionalized donor and acceptor monomers. It can be expected that the crystal structure and pore size of the COFs will not change when just the orientation of the imine linkage is reversed, which is also a prerequisite to discuss the effects of constitutional isomerism. Some of the finally prepared six COFs (three constitutional isomer pairs) have been reported already and are synthetically accessible under mild solvothermal conditions^18,33^. For the COFs not described in the literature, synthetic conditions were optimized to yield a high surface area with good crystallinity (for synthetic details, see Supplementary Note 2). The successful synthesis of the isomeric COFs was confirmed by a combination of techniques. Powder X-ray diffraction (PXRD) measurements were first conducted to confirm the crystal structure of the COFs. In all cases, constitutionally isomeric COFs DCNA and DNCA showed identical experimental PXRD patterns, which also match well with the simulated PXRD patterns (Fig. 2c, Supplementary Figs. 1, 2) of the 2D honeycomb (**hcb**) topology. In addition, the intense and sharp diffraction peaks indicated their high crystallinity. The chemical structure of DCNA and DNCA COFs was further investigated by Fourier transform infrared spectroscopy (FTIR) (Fig. 2d, Supplementary Fig. 3) and ^13^C cross-polarization magic angle spinning (CP-MAS) solid-state NMR (Supplementary Fig. 4). In the FTIR, the characteristic signal of the –C = N– stretching appeared at different wavenumbers for COF DCNA-1 (1625 cm^−1^) and DNCA-1 (1620 cm^−1^), showing that the imine linkages have a different chemical environment. On the other hand, the characteristic peaks of the triazine (stretching bands ~1502 cm^−1^, ~1360 cm^−1^; breathing mode: ~809 cm^−1^)^17,34^ appeared at almost identical wavenumbers (Fig. 2d, Supplementary Fig. 3). In the ^13^C CP-MAS solid-state NMR spectroscopy measurement, again, the signal assigned to the imine carbon atoms of DCNA-1 and DNCA-1 are slightly shifted, namely located at 159 and 156 ppm, respectively, while the chemical shift of the triazine carbons is identical at 170 ppm for both isomers (Supplementary Fig. 4). Besides, the signal of the carbon atoms neighboring the imine nitrogen are found at 152 ppm and 145 ppm for DCNA-1 and DNCA-1, respectively. This further proves the distinct chemical environment of the imine linkages of the constitutional isomers DCNA-1 and DNCA-1. The permanent porosity of the COFs was evaluated by nitrogen (N_2_) sorption. DCNA-1 showed a Brunauer–Emmett–Teller (BET) surface area of 1066 m^2^ g^−1^, whereas DNCA-1 showed a BET surface area of 456 m^2^ g^−1^, pointing to a slightly less ordering in the latter. Also, DCNA-2 shows a higher surface area than DNCA-2, while the opposite is seen for the DCNA/DNCA-3 pair (Supplementary Fig. 5). The difference in surface area of the COFs can be attributed to the varied reactivity and solubility of the monomers used to synthesize each COF isomer, which leads to slight differences in the degree of order of the COFs. Scanning electron microscopy (SEM) measurements show an irregular morphology for all COFs (Supplementary Fig. 6). Overall, it can be stated that all DCNA/DNCA COF pairs are structural equivalents, with only the –C=N– linkage pointing in opposite directions besides differences in the accessible surface areas.

### Photophysical behavior of the pristine isomeric COFs

While it is seen that the change in –C=N– linkage orientation is not changing the topology of the COFs, the subtle change in chemical structure has a profound influence on the optoelectronic properties of the COFs. This can be already easily spotted by eyesight, as DCNA-1 is a yellow, while DNCA-1 is a dark-red powder (Insets of Fig. 2e), which is also reflected by the absorption edge in the ultraviolet-visible diffuse reflectance (UV–Vis DRS) spectra. DCNA-1 and DNCA-1 both exhibit broad light absorption with the absorption onsets located at 520 nm and 645 nm, respectively (Fig. 2e). Thus, a shift of the absorption onset of more than 120 nm is induced by inverting the imine linkage. Accordingly, the optical bandgaps calculated from the corresponding Tauc plots are 2.48 eV for DCNA-1 and 2.06 eV for DNCA-1 (Supplementary Fig. 8). The same trend, even though not such pronounced, is observed for the other COF isomers DCNA/DNCA-2 and -3 (Supplementary Figs. 7, 8). In general, DNCAs show light absorption up to higher wavelengths, thus smaller optical bandgaps. The exciton and charge carrier dynamics of the COFs were probed by time-resolved spectroscopies. Photoconductivity of the COFs was measured by flash-photolysis time-resolved microwave conductivity (FP-TRMC) spectroscopy. FP-TRMC is a rapid electrodeless method to evaluate the intrinsic/local charge-transporting property of semiconductors, which provides a measure of the photoconductivity as ΦΣ*μ*, where Φ and Σ*μ* correspond to the photocarrier generation yield and the total charge carrier mobility (combined electron and hole mobility), respectively^35^. Maximum photoconductivities of 4.0 × 10^−6^ and 2.0 ×10^−6^ cm^2^ V^−1^ s^−1^ were observed for DCNA-1 and DNCA-1, respectively, revealing higher photoconductivity of DCNA-1 compared to DNCA-1 (Fig. 2f). Electron paramagnetic resonance (EPR) conduction band (CB) spectra of the COFs were further measured in the dark and under light irradiation to gain an insight into their charge separation and transfer processes (Fig. 2g,h). The COFs exhibited a relatively small EPR signal at *g* = 2.005, indicating the presence of unpaired electrons already in the dark state^36^. After switching on the light, the EPR signal intensity increases significantly for all COFs, indicating that more electrons were excited from the valence band (VB) to the CB. Upon light irradiation, DNCA-1 has a higher EPR signal intensity. The higher EPR intensity can also be attributed to the relatively better light absorption ability of DNCA-1. However, DNCA-1 showed just a ~33% increased light absorption (calculated by integrating the UV-Vis DRS spectra) compared to DCNA-1 (Fig. 2e), which is much lower than the ~500% stronger EPR signal intensity (Fig. 2g, h), not to mention that the ~33% light absorption enhancement can only partially contribute to the enhanced EPR signal. Thus, DNCA-1 indeed shows relatively better charge separation efficiency compared to DCNA-1. However, the EPR signal reaches the steady state much faster for DCNA-1 compared to DNCA-1 upon light irradiation, which indicates a faster charge separation and lower trapping of charge carriers in DCNA-1. From these measurements, it can be concluded that DNCA-1 exhibits a broader light absorption, thus a smaller optical bandgap and higher charge separation efficiency than DCNA-1. On the other hand, DCNA-1 possesses faster charge separation and higher photoconductivity than DNCA-1. Thus, even though the COFs are constructed from identical organic building blocks, reversing the orientation of their connection, i.e., of the imine linkages, triggers distinct changes in their optoelectronic properties.

### Photophysical behavior of the protonated isomeric COFs

**Fig. 3:**
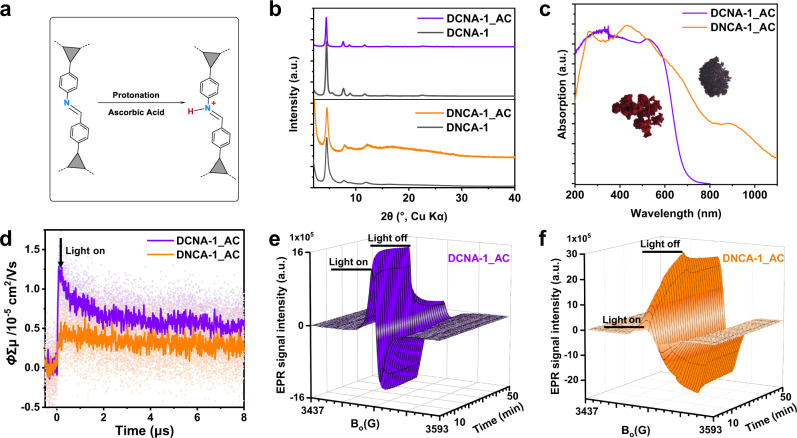
Photophysical properties of the protonated isomeric COFs. **a** Scheme of the protonation of the imine linkage. **b** PXRD patterns, (**c**) UV-Vis DRS spectra, (**d**) FP-TRMC transients of DCNA-1_AC and DNCA-1_AC. EPR CB electrons spectra of DCNA-1_AC (**e**) and DNCA-1_AC (**f**) during and after visible light irradiation (>420 nm, 300 W Xe lamp). Insets of (**c**) show the corresponding photographs of DCNA-1_AC (dark red) and DNCA-1_AC (dark brown).

Our previous work has shown the superior photocatalytic performance of imine-based COFs when the imine linkages are protonated^18^. Therefore, also the protonated forms of all constitutional isomers were considered (Fig. 3, Supplementary Note 4). The protonated forms of the COFs are named DCNA-*X*_AC and DNCA-*X*_AC, respectively, as ascorbic acid (AC) was used as acid for protonation, which also acts as the sacrificial reductant in the photocatalytic hydrogen production experiments shown later. AC protonated COFs were prepared via a 5-minute treatment in 0.1 mol/L AC aqueous solution followed by filtration and drying overnight by ultra-high vacuum at room temperature. The protonation of imine moieties was further confirmed by FTIR measurements, from which the imine peaks at ~1620 cm^−1^ disappeared, and new bands appeared at ~1790 cm^−1^ (Supplementary Fig. 10). After protonation, relative intensity changes in the reflections in the PXRD patterns were observed due to the presence of ascorbate anions within the pores (Fig. 3b, Supplementary Fig. 9). The inclusion of ascorbate anions is also seen by the vastly decreased N_2_ adsorption (Supplementary Fig. 11). After deprotonation, PXRD patterns similar to the pristine COFs are observed, showing that the ascorbate anions can be reversibly removed from the pores (Supplementary Fig. 9). The protonation degree was calculated based on high-resolution X-ray photoelectron spectroscopy (XPS) (Supplementary Table 1).

In the next step, the photophysical properties of the isomeric protonated COFs DCNA-X_AC and DNCA-X_AC were investigated and compared (The comparison of the COFs before and after protonation is shown in Supplementary Note 5). As seen from the UV-Vis DRS spectra, also in the protonated forms, the linkage orientation induces significant changes in the absorption properties. Again, a red-shift of absorption is observed from the DCNA to the DNCA COFs. Thus DCNA-1_AC is dark-red, while DNCA-1_AC is a dark-brown powder (Insets of Fig. 3c). DCNA-1_AC and DNCA-1_AC both exhibit broad light absorption with bandgap absorption onsets located at 685 and 916 nm, respectively (Fig. 3c). Thus, a shift of the absorption onset of 231 nm is induced by inverting the protonated imine linkage. Besides, for DNCA-1_AC another less intense absorption in the near IR region is observed. Accordingly, the bandgaps calculated from the corresponding Tauc plots are 1.90 eV for DCNA-1_AC and 1.56 eV for DNCA-1_AC (Supplementary Fig. 13). The same trend was observed for the other isomeric COF pairs DCNA/DNCA-2_AC and DCNA/DNCA-3_AC, even though not such pronounced (Supplementary Figs. 12, 13). DCNA-2_AC and DCNA -3_AC show light absorption onsets at 689 and 593 nm, with a calculated optical bandgap of 1.90 and 2.28 eV, respectively. In contrast, DNCA-2_AC and DNCA -3_AC show light absorption onsets at 874 and 639 nm, with a calculated bandgap of 1.65 and 2.12 eV, respectively. In general, the DNCA_AC COFs show a broader light absorption, thus smaller optical band gaps than the DCNA_AC COFs, as was observed for the unprotonated forms. Afterward, the photoconductivity of the DCNA-1_AC and DNCA-1_AC was evaluated by using FP-TRMC measurements (Fig. 3d, Supplementary Fig. 15). Maximum photoconductivities of 1.3 × 10^−5^ and 6.0 × 10^−6^ cm^2^ V^−1^ s^−1^ were observed for DCNA-1_AC and DNCA-1_AC, respectively. These results revealed the more efficient charge-transporting ability of DCNA-1_AC compared to DNCA-1_AC, as well as other reported COFs with values usually measured to be in the 10^−6^ cm^2^ V^−1^ s^−1^ range^35,37^. EPR CB electron spectra were also measured for DCNA-1_AC and DNCA-1_AC. The EPR intensity of DNCA-1_AC was ~2 times higher than for DCNA-1_AC (Fig. 3e, f, Supplementary Fig. 16). Again, that only the broader light absorption induced higher EPR intensity can be excluded, as less than 50% light absorption enhancement of DNCA-1_AC contributes to the enhanced EPR signal. These significant differences in the constitutionally isomeric COFs should also strongly influence their performance in photochemical or -catalytic applications.

### Photocatalytic HER performance of the isomeric COFs

**Fig. 4:**
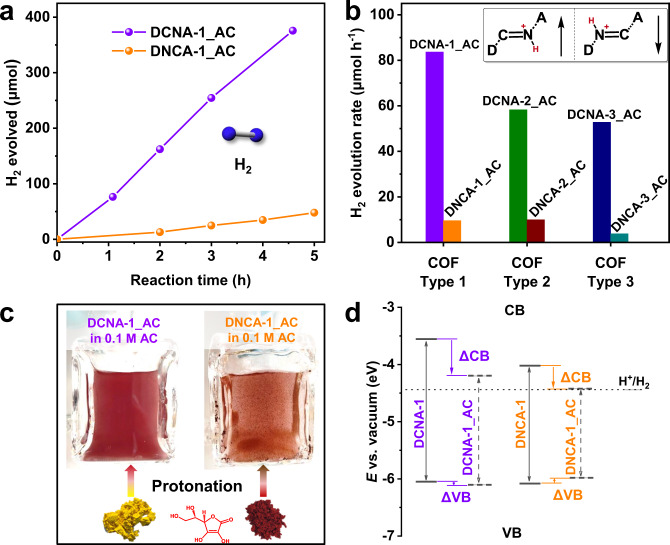
Photocatalytic HER performance of the isomeric COFs. **a** The HER evolution of the isomers DCNA-1_AC and DNCA-1_AC using AC as sacrificial electron donor (3 mg catalyst, 16 mL 0.1 mol L^−1^ (M) AC aqueous solution, 3 µL H_2_PtCl_6_ (8 wt.% in water), λ >  420 nm, 20 °C). **b** Comparison of the photocatalytic HER rates of all DCNA_AC and DNCA_AC COFs studied in this project. **c** Digital photographs of the protonation process of DCNA-1 and DNCA-1 in 0.1 M ascorbic acid aqueous solution. **d** Experimental band structures of the isomeric COFs DCNA-1 and DNCA-1 and their protonated counterparts DCNA-1_AC and DNCA-1_AC.

To investigate the impact of the changing optoelectronic properties of constitutionally isomeric COF pairs on their photocatalytic performance, photocatalytic hydrogen evolution reaction (HER) from water with all COFs as photocatalysts was investigated^38–40^. The reactions were performed under visible light irradiation (λ >  420 nm) and in the presence of 0.1 M AC (pH = 2.50, room temperature) as the sacrificial electron donor (SED) and Pt as co-catalyst. As expected, an immediate and obvious darkening of the color of the pristine COFs was observed when they were dispersed in the reaction solutions, indicating that the actual catalysts for photocatalysis are the protonated COFs (Fig. 4c, Supplementary Fig. 17). As expected from the measured optical and electronic properties, the isomeric COFs DCNA_AC and DNCA_AC showed remarkably different photocatalytic HER activity (Fig. 4a, b). DCNA-1_AC, with the donor moiety linked to the imine carbon and acceptor moiety linked to the imine nitrogen in the backbone, showed an HER rate of 83.66 ± 2.27 μmol h^−1^ (corresponding to 27.9 mmol h^−1^ g^−1^, see Supplementary Table 3). In contrast, DNCA-1_AC with the opposite orientation of the imine linkage just produced H_2_ with a rate of 9.57 ± 0.82 μmol h^−1^ (Fig. 4a). The same trend was also observed for DCNA-2_AC/DNCA-2_AC and DCNA-3_AC/DNCA-3_AC (Fig. 4b, Supplementary Table 3). DCNA-2_AC and DCNA−3_AC produced H_2_ with a rate of 58.25 and 52.81 μmol h^−1^, respectively, whereas DNCA-2_AC and DNCA−3_AC showed HER rates of 9.94 and 3.78 μmol h^−1^, respectively. Likewise, the photocatalytic HER was also conducted under alkaline conditions by using triethanolamine (TEOA) (pH = 10.65, room temperature) as SED in which the COFs retained their pristine chemical structures (Supplementary Fig. 18). However, neither DCNA nor DNCA COFs can evolve a detectable amount of H_2_ (Supplementary Fig. 19) under these conditions. This is consistent with other reports and further proves that the protonation of the imine bond facilitates photocatalytic HER^18,41,42^. Finally, the apparent quantum efficiency (AQE) at 420 nm of DCNA-1_AC was measured to be 1.49 %, while the AQE of DNCA-1_AC was too low to be accurately measured.

Photocatalysis involves 3 key steps: (1) light absorption and exciton generation, (2) charge carrier separation, transport, and recombination, and finally (3) surface reaction. Based on the optoelectronic properties observed before, DNCA-1_AC possesses broader light absorption (Step 1) and better charge separation efficiency (Step 2), which both should contribute to better photocatalytic performance. However, the photocatalytic results contradicted these observations. This led us to focus on the surface reaction step (Step 3) of the photocatalysis process. Valence band XPS (VB-XPS) was applied to detect the VB positions of all COFs before and after protonation (Supplementary Fig. 20). Combining the VB positions and the bandgaps, the band structures of the COFs were obtained (Fig. 4d, Supplementary Fig. 21, Supplementary Table 4)^43^. The CB value of DCNA-1_AC decreased by 0.64 eV (∆CB) upon protonation, but its VB value is almost identical to the pristine COF DCNA-1. Similarly, DNCA-1_AC shows a decrease of CB position by 0.40 eV, and its VB position increased slightly by 0.1 eV (∆VB). Thus, protonation significantly decreased the CB positions of the COFs but did not change their VB positions much, which was also observed for the other two DCNA/DNCA pairs and their protonated counterparts (Supplementary Fig. 21). For DNCA-1_AC, its CB position is almost the same as the hydrogen production energy level. Considering the reaction overpotential as well, the CB electrons of DNCA-1_AC can hardly reduce H^+^ to H_2,_ which might lead to the much lower H_2_ evolution rate of DNCA-1_AC. In comparison, DCNA-1_AC possesses a higher CB position which yields a stronger reduction potential *vs. E*(H^+^/H_2_) and finally enables the fast photocatalytic HER. In addition, the greater charge carrier mobility of DCNA-1_AC compared to DNCA-1_AC should facilitate the charge transfer to the metal nanoparticles as active sites for proton reduction, thus contributing to the better activity of DCNA-1_AC as well (Fig. 3d).

After photocatalysis, the used COFs were recovered and characterized. All the COFs remained their pristine chemical structures as the imine peaks can be seen from the FTIR spectra (Supplementary Fig. 22). A decreased crystallinity was observed from the PXRD patterns for all recovered DCNA COFs. In contrast, all recovered DNCA COFs almost lost their structural order (Supplementary Fig. 23). The better stability of DCNA COFs compared to DNCA COFs could also contribute to the better performance of DCNA COFs during photocatalysis. Finally, it should be mentioned that the different crystallinity and porosity of the pristine COFs are not main factors for their photocatalytic performance. For example, the crystallinity and porosity of DCNA-3 are lower than those of DNCA-3, but DCNA-3_AC still showed much better photocatalytic HER activity than DNCA-3_AC (Fig. 4b, Supplementary Figs. 2–5).

### Density functional theory calculations of the COFs

**Fig. 5:**
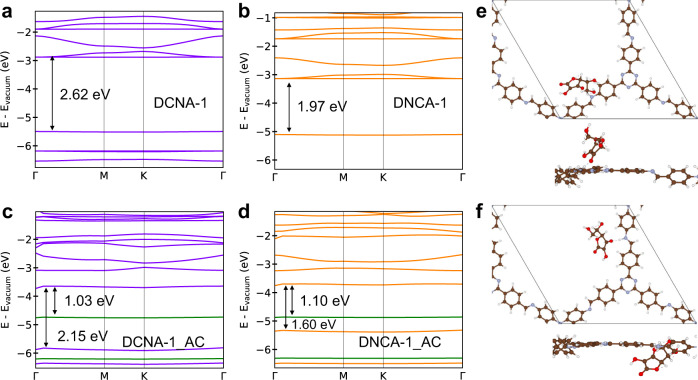
Density functional theory calculations of the COFs. Calculated HSE electronic band structures of DCNA-1 (**a**), DNCA-1 (**b**) and their protonated forms DCNA-1_AC (**c**), DNCA-1_AC (**d**). The green lines indicate states of the ascorbate anion. **e**, **f** Periodic structure models of DCNA-1_AC and DNCA-1_AC. The ascorbate anion was placed in the protonated imine group, creating a hydrogen bond. The introduction of one ascorbate anion and one proton in the unit cell means that 1/3 of the imine sites are protonated. C, N, O, and H are shown in brown, blue, red, and white, respectively.

To further unravel the impact of constitutional isomerism, density functional theory (DFT) calculations were performed on the isomeric COFs DCNA-1/DNCA-1 and their protonated forms DCNA-1_AC/DNCA-1_AC (Fig. 5). Using periodic one-layer models, the geometry of the chemical structures, including the ascorbate anion in the case of DCNA-1_AC and DNCA-1_AC, were optimized on the level of gradient-corrected DFT using the PBE functional, and the band structures were computed using the accurate Heyd-Scuseria-Ernzerhof (HSE) functional which contains exact exchange (Figs. 5a–d). The HSE band structures show dispersion-less valence and conduction bands for all COFs. As seen, the calculated values are in reasonable agreement with experimental values (Supplementary Table 5). From the DFT calculation, the bandgap of DCNA-1 (2.62 eV) is larger than that of DNCA-1 (1.97 eV), and protonation reduces the bandgap to 2.15 eV for DCNA_1-AC and 1.60 eV for DNCA-1_AC (Fig. 5a–d, Supplementary Fig. 31). The HOMO-LUMO gaps using cluster models with the CAM-B3LYP functional are much larger but show the same trend (Supplementary Table 6). For a more direct comparison of the UV-vis spectra, we calculated transition energies and probabilities using time-dependent DFT (TD-DFT). The lowest excitation energies with non-vanishing oscillator strength are 3.39 eV and 3.18 eV for DCNA-1 and DNCA-1, respectively. The excitation energies of the corresponding isomeric protonated COFs DCNA-1_AC and DNCA-1_AC are 2.42 eV and 2.03 eV, respectively (Supplementary Table 6). Analysis of Natural Transition Orbitals (NTOs) shows a slightly higher degree of delocalization for DNCA-1 compared to DCNA-1 (Supplementary Figs. 25–30). Besides, the NTOs of the isomeric DCNA-1 and DNCA-1 are very similar, and their isostructural protonated forms DCNA-1_AC and DNCA-1_AC are also similar. The NTOs indicate that the excitations involve a small amount of charge transfer. We also calculated from periodic HSE models the valence band maximum (VBM) and conduction band minimum (CBM) relative to the vacuum level (Supplementary Table 5). In particular, the CBM shifts to lower energies upon protonation for both isostructural COFs, while the VBM is less affected. This further corroborates the experimental band structures (Fig. 4d). The calculated CBM shift (∆CB) is -0.85 eV for DCNA-1/ DCNA-1_AC and −0.62 eV for DNCA-1/DNCA-1_AC, respectively, compared to the ∆CB of −0.64 and −0.40 eV in experiments.

### Scope of the theory

**Fig. 6:**
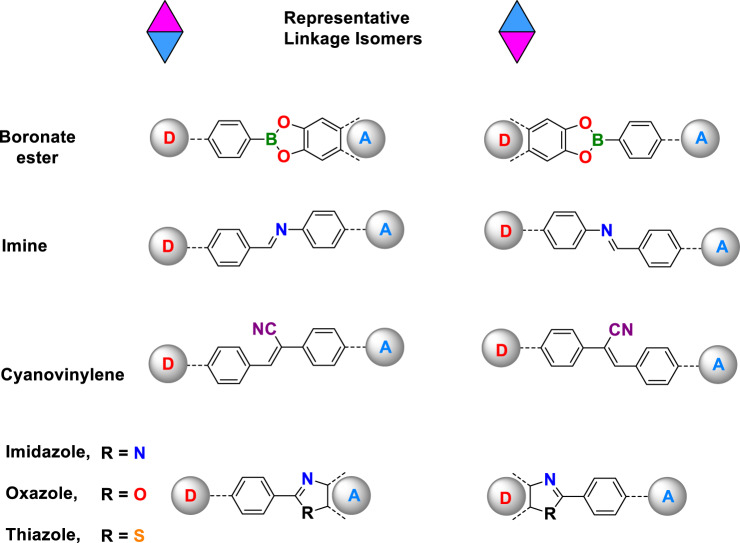
Scope of the theory. Representative linkages which show constitutional isomerism in COFs.

The outstanding feature of COFs, distinguishing them from conventional organic polymer networks, is their crystallinity. The crystallization of COFs is usually gained by applying reversible covalent bonds, which normally consist of heteroatoms. Consequently, the formed linkages mostly have constitutional isomers depending on the position of the building components. Thus, besides imine linkages, the concept of linkage isomerism can probably be extended to broader linkage scopes in COFs synthesis such as boronate ester^1^, hydrazone^15^, imide^44^, amide^20^, cyanovinylene^45^, amine^21^, oxazole^22^, thiazole^22,46^, or imidazole^47^ linkages (Fig. 6, Supplementary Fig. 32). It can be anticipated that the application potential of COFs will be further exploited by also focusing on the constitutional isomerism of the linkages.

## Discussion

In conclusion, we propose that the constitutional isomerism of the linkages, i.e., the orientation of chemical connections towards the COF building blocks, should be considered and treated with the same importance as the nature of the backbone functionalities when designing COFs. The imine linkage, as the most reported linkage in COF chemistry so far, was chosen as a representative to elucidate the effect of linkage isomerism in donor–acceptor COFs. Two kinds of constitutionally isomeric COFs were synthesized, namely DCNA (Donor-C = N-Acceptor) and DNCA (Donor-N = C-Acceptor) COFs with identical functionalities but opposite orientations of the imine linkage against the donor and acceptor motif. The linkage isomerism has a distinct impact on the photophysical properties of the constitutionally isomeric and isoreticular COFs. Consequently, COFs with different linkage orientations show distinct differences in their performance as photocatalysts for the HER from water. HER rates as high as 83.66 μmol h^−1^ (27.9 mmol h^−1^ g^−1^), can be achieved for the strongest donor–acceptor combination. Experimental results and DFT simulation both point to the crucial influence of imine linkage orientation on bandgap and especially CBM to explain the different catalytic performances. This work shows that constitutional isomerism is a fundamental aspect to understand COF properties and performances. It is plausible that this concept can also be extended to various other linkages in COFs.

## Methods

### COF synthesis

Generally, all the COFs were synthesized by the solvothermal method in a Pyrex tube at 120 °C for 3 days. A Pyrex glass tube (15 mL) was charged with the donor and acceptor monomers, specific solvents, and 6 M acetic acid aqueous solution. The tube was first sonicated until a fluffy solid formed and then flash frozen at 77 K (liquid N_2_ bath) and degassed by three times freeze-pump-thaw cycles. The internal pressure was evacuated to 10^−3^ mbar. Then, the tube was sealed and heated at 120 °C for 3 days. The final fluffy precipitate was filtered and washed with acetone several times. Finally, the COF powders were dried in a normal oven at 80 °C. The specific combinations of the solvents are given in Supplementary Note 2.

### Photocatalytic hydrogen evolution reactions

The photocatalysis was conducted in a 36 mL side irradiation quartz reactor equipped with a 152 mL glass gas container. Generally, 3 mg pristine COF were dispersed in either 16 mL 0.1 M AC aqueous or triethanolamine (TEOA)/water (v:v = 2 mL: 16 mL) solution. 3 microliter H_2_PtCl_6_ aqueous solution (8 wt%) were added as the source of Pt co-catalyst. The reactor was sealed with rubber stoppers and degasses by Argon for 30 minutes before irradiation. Then, the reactor was irradiated with a 300 W Xe lamp (L.O.T-Quantum design) with appropriate filters. The temperature of the system was kept at 20 °C by water circulation. The gas sample was taken from the headspace, and H_2_ was quantified by GC (Agilent 7820 A) equipped with a thermal conductivity detector. The photocatalytic hydrogen evolution reaction rates in AC were determined from the linear regression fit of the H_2_ evolution curves, and the pressure increase was neglected in the calculations. Due to the negligible amount of H_2_ evolved by DNCA/DNCA-X_AC COFs, H_2_ was only measured after finishing the irradiation. All the reacted COFs were recovered by filtration, washed with acetone, and dried at 80 °C overnight for further characterization.

### Quantum chemical calculations

Calculations on periodic models were performed with VASP^48,49^, version 5.4.4. Atomic positions and cell parameters were optimized using the PBE functional^50^, while band structures were calculated using HSE^51,52^. The D3 dispersion correction^53^ with Becke-Johnson damping was used^54^. The kinetic energy cutoff was set to 600 eV, and a 2×2×1 **k** point grid was used. The counterion for calculations on the protonated forms was ascorbate. The cell vector perpendicular to the layer was set to 20 Å, resulting in a vacuum space of at least 12 Å for the protonated COFs. Elementary cells are shown in Supplementary Fig.24.

Cluster calculations were performed using ORCA^55^, version 4.0.0.2. The cluster model was cut out of the periodic structures and contained one whole pore (i.e., three donor and three acceptor units). Only the protonated imine groups were optimized, while all other atoms were kept fixed. This optimization was done with PBE functional^50^ and with the D3 dispersion correction^53^ with Becke–Johnson damping^54^, using the 6-311G** basis set^56^. CAM-B3LYP^57–60^ calculations were performed using the def2-SVP basis set^61^, which gives very similar results compared to the larger 6-311G** basis set. Time-dependent density functional theory (TD-DFT)^62^ was used to calculate excitation energies and oscillator strengths. Natural transition orbitals (NTOs)^63^ were used to analyze the electron-hole pairs contributing to the excitations.

## Supplementary information

Supplementary Information

## Data Availability

All data are available in the main text or the supplementary information. Source data are provided with this paper.

## Source data

Source data
