# Supplementary material for: Constitutional isomerism of the linkages in donor–acceptor covalent organic frameworks and its impact on photocatalysis
Source: Nat Commun. 2022 Oct 23;13:6317. doi: 10.1038/s41467-022-33875-9 (PMC9588771; doi:10.1038/s41467-022-33875-9)
Supplement: Supplementary file 1 — Supplementary Information [file 41467_2022_33875_MOESM1_ESM.pdf]

Constitutional isomerism of the linkages in donor-acceptor covalent organic frameworks and its impact on photocatalysis

Table of Contents

Supplementary Note 1. Materials and methods

Supplementary Note 2. Syntheses of the isomeric COFs

Supplementary Note 3. Characterization of the pristine COFs

Supplementary Note 4. Characterization of protonated COFs

Supplementary Note 5. Comparison of pristine and protonated COFs

Supplementary Note 6. Photocatalytic H_2 evolution

Supplementary Note 7. Quantum chemical calculations

Supplementary Note 1. Materials and methods

1.1 Materials

All the reagents and solvents used for the synthesis were commercially available and used without further purification. The 1,4-dioxane (99.9%), mesitylene (1,3,5-trimethylbenzene, >98%) o-DCB (1,2-Dichlorobenzene, 99%) anhydrous n-BuOH (n-Butanol, 99%) were purchased from Sigma Aldrich Chemicals. 2,4,6-Tris(4-aminophenyl)triazine, 95%, Tris(4-formylphenyl)amine, >97.0%, 1,3,5-Tris(4-formylphenyl)benzene, >96.0% and 1,3,5-Tris(4-aminophenyl)benzene, >93.0% were all supplied by TCI. Tris(4-aminophenyl)amine 98% was purchased from ABCR. Acetic acid (>99.0%) was purchased from Carl Roth.

1.2 Characterization

1.2.1 Powder X-ray diffraction (PXRD) analysis

Powder X-ray diffraction data were collected on a Bruker D8 Advance diffractometer in reflection geometry operating with a Cu Kα anode (λ = 1.54178 Å) operating at 40 kV and 40 mA. Samples were ground and mounted as loose powders onto a Si sample holder. PXRD patterns were collected from 2 to 60 2θ degrees with a step size of 0.02 degrees and an exposure time of 2 seconds per step.

1.2.2 Fourier transform infrared spectroscopy (FTIR) analyses

The Fourier transform infrared spectroscopy (FTIR) analyses of the samples were carried on a Varian 640IR spectrometer equipped with an ATR cell.

1.2.3 Solid-state diffuse reflectance Ultraviolet-visible spectroscopy (UV-Vis DRS) analysis

Solid-state UV-Vis DRS spectra of the COF powders have been collected on Varian Cary 300 UV-Vis Spectrophotometer.

1.2.4 N_2 physisorption measurements:

N_2 sorption measurements of the pristine COFs were performed on a volumetric sorption instrument (Autosorb-iQ-MP). Before the gas sorption studies of COFs, the samples were dried under a dynamic vacuum (<10^{-3} Torr) at room temperature (RT) followed by heating to 120 °C for 12 h. Using the N_2 adsorption isotherms, the surface areas were calculated over a pressure range 0.01-0.9 = p/p_0 using Brunauer-Emmett-Teller (BET), and pore size distributions were calculated using the quenched solid density functional theory (QSDFT) or non-local density functional theory (NLDFT) method on the N_2 adsorption branch. N_2 sorption measurements of the protonated COFs were performed on Quadrasorb instrument.

1.2.5 Scanning electron microscope (SEM)

The SEM analyses of COF samples were performed on an S-2700 scanning electron microscope (Hitachi, Tokyo, Japan).

1.2.6 Electron paramagnetic resonance spectroscopy (EPR)

EPR measurements in X-band (microwave frequency ≈ 9.87 GHz) were performed at 293 K by a Bruker EMX CW micro spectrometer equipped with an ER 4119HS-WI high-sensitivity optical resonator with a grid on the front side. The samples were illuminated by a 300 W Xe lamp with a 420 nm cutoff filter (LOT Oriel). All the samples were measured under the same conditions (microwave power: 6.74 mW, modulation frequency: 100 kHz, modulation amplitude: 3 G, Sweep time: 45 s). g values have been calculated from the resonance field B_0 and the resonance frequency ν using the resonance condition $h\nu = g\beta B_0$. The calibration of the

g values was performed using DPPH (2, 2-diphenyl-1- picrylhydrazyl) (g = 2.0036 ± 0.00004).

1.2.7 Flash-photolysis time-resolved microwave conductivity (FP-TRMC) spectroscopy

The charge carrier transport property was evaluated by the FP-TRMC technique at room temperature under an N_2 atmosphere. Transient charge carriers were generated through photoexcitation by laser pulses of third-harmonic generation (λ = 355 nm) from a Spectra-Physics INDI–HG Nd: YAG laser with a pulse duration of 5 - 8 ns at the photon density of 4.6 × 10^{15} photon cm^{-2}. The frequency and power of the probing microwave were set at around 9.1 GHz and 3 mW, respectively, so that the electric field of the microwave was sufficiently small not to disturb the motion of charge carriers. Photoconductivity transients demodulated through a GaAs crystal-diode with Schottky-barriers (rise time < 1 ns) were monitored by a Tektronix model TDS3032B digital oscilloscope. The time constant (τ) of the present TRMC system was then determined by the Q-value of microwave cavity (Q = 2000), leading to $\tau = Q/2f \sim 100$ ns. The observed conductivities were normalized, given by a photocarrier generation yield (Φ) multiplied by the sum of the carrier mobility of electron/hole ($\Sigma\mu$), according to the equation, $\Phi\ \Sigma\mu = A\Delta P_r/eI_0F_LP_R$ where, e, A, I_0 F_L, P_R, and $\Delta\ P_r$ are elementary charge, sensitivity factor (S cm^{-1}), incident photon density of the excitation laser (photon cm^{-2}), a correction factor (cm^{-1}) for overlapping between the special distribution of photo-generated charge carriers and electromagnetic field strength of probing microwave in the cavity, and reflected microwave power and its transient change, respectively.

1.2.8 X-ray photoelectron spectroscopy (XPS)

XPS was measured on K-Alpha™ + X-ray Photoelectron Spectrometer System (Thermo Scientific) with Hemispheric 180° dual-focus analyzer with 128-channel detector. X-ray monochromator is Micro focused Al-Kα radiation. For the measurement, the prepared powder samples were pressed and loaded on carbon taps, then pasted onto the sample holder for measurement. The data was collected with an X-ray spot size of 400 µm, 20 scans for the survey, 50 scans for the specific regions, and 100 scans for the valance band (VB) regions.

1.3 Theoretical calculations

Calculations on periodic models were performed with VASP[1,2], version 5.4.4. Atomic positions and cell parameters were optimized using the PBE functional,[3], while band structures were calculated using HSE.[4,5] The D3 dispersion correction[6] with Becke-Johnson damping was used.[7] The kinetic energy cutoff was set to 600 eV, and a 2×2×1 k point grid was used. The counterion for calculations on the protonated forms was ascorbate. The cell vector perpendicular to the layer was set to 20 Å, resulting in a vacuum space of at least 12 Å for the protonated COFs. Elementary cells are shown in Supplementary Fig.24.

Cluster calculations were performed using ORCA,[8] versions 4.0.0.2. The cluster model was cut out of the periodic structures and contained one whole pore (i.e., three donor and three acceptor units,). Only the protonated imine groups were optimized, while all other atoms were kept fixed. This optimization was done with PBE functional[3] and with the D3 dispersion correction[6] with Becke-Johnson damping,[7] using the 6-311G** basis set.[9] CAM-B3LYP[10–13] calculations were performed using the def2-SVP basis set,[14], which gives very similar results compared to the larger 6-311G** basis set. Time-dependent density functional theory (TD-DFT)[15] was used to calculate excitation energies and oscillator strengths. Natural transition orbitals (NTOs)[16] were used to analyze the electron-hole pairs contributing to the excitations.

Supplementary Note 2. Syntheses of the isomeric COFs

Generally, all the COFs were synthesized by the solvothermal method in a Pyrex tube at 120 °C for 3 days.

2.1 Synthesis of DCNA-1[17]:

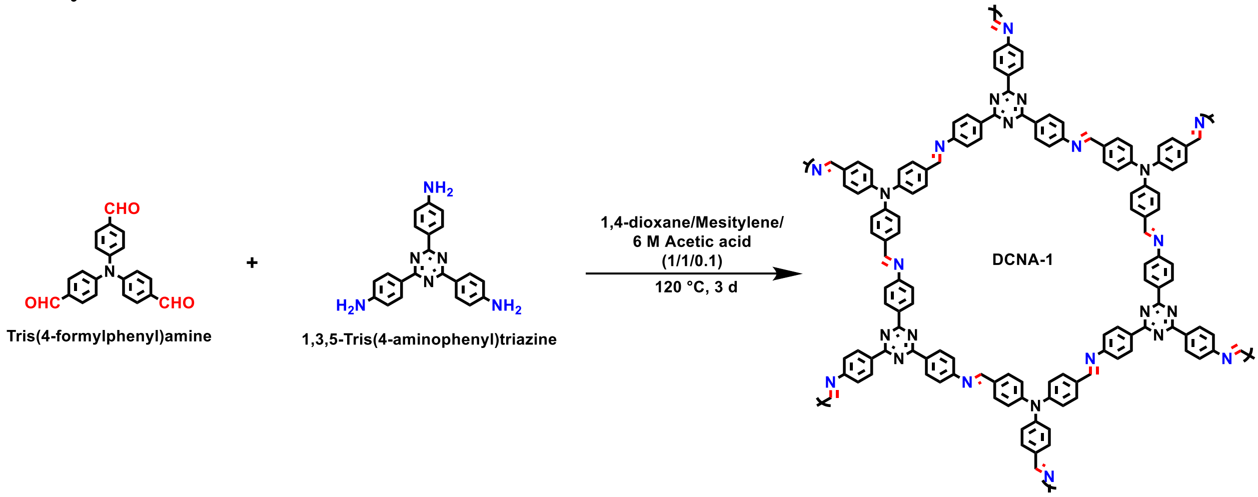

A Pyrex glass tube (15 mL) was charged with Tris(4-formylphenyl)amine (Donor) (65.9 mg, 0.2 mmol), 1,3,5-Tris(4-aminophenyl)triazine (Acceptor) (70.9 mg, 0.2 mmol), 3 mL dioxane, 3 mL mesitylene and 0.3 mL 6 M acetic acid aqueous solution. The tube was first sonicated until a fluffy solid formed and then flash frozen at 77 K (liquid N_2 bath) and degassed by three times freeze-pump-thaw cycles. The internal pressure was evacuated to 10^{-3} mbar. The tube was sealed and heated at 120°C for 3 days. The final bright yellow precipitate was washed with acetone several times and collected by filtration. Finally, the DCNA-1 powder was dried in a normal oven at 80 °C. Yield = 79.4 % (100.0 mg)

2.2 Synthesis of DNCA-1[18]:

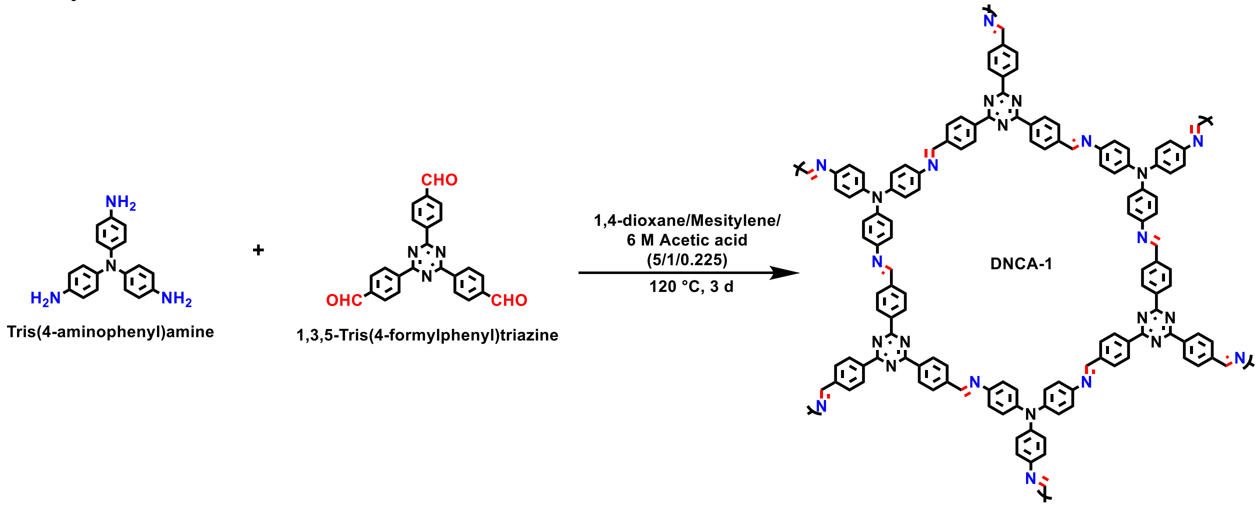

A Pyrex glass tube (15 mL) was charged with Tris(4-aminophenyl)amine (Donor) (29.2 mg, 0.1 mmol), 1,3,5-Tris(4-formylphenyl)triazine (Acceptor) (39.3 mg, 0.1 mmol), 4 mL binary solvent (1,4-dioxane : Mesitylene = 5:1) and 0.15 mL 6 M acetic acid aqueous solution. The tube was first sonicated to form a red fluffy solid and then flash frozen at 77 K (liquid N_2 bath) and degassed by three times freeze-pump-thaw cycles. The internal pressure was evacuated to 10^{-3} mbar. The tube was sealed and heated at 120 °C for 3 days. The final orange-red precipitate was washed with acetone several times and collected by filtration. Finally, the DNCA-1 powder was dried in a normal oven at 80 °C. Yield = 95.1% (60.0 mg)

2.3 Synthesis of DCNA-2[17]:

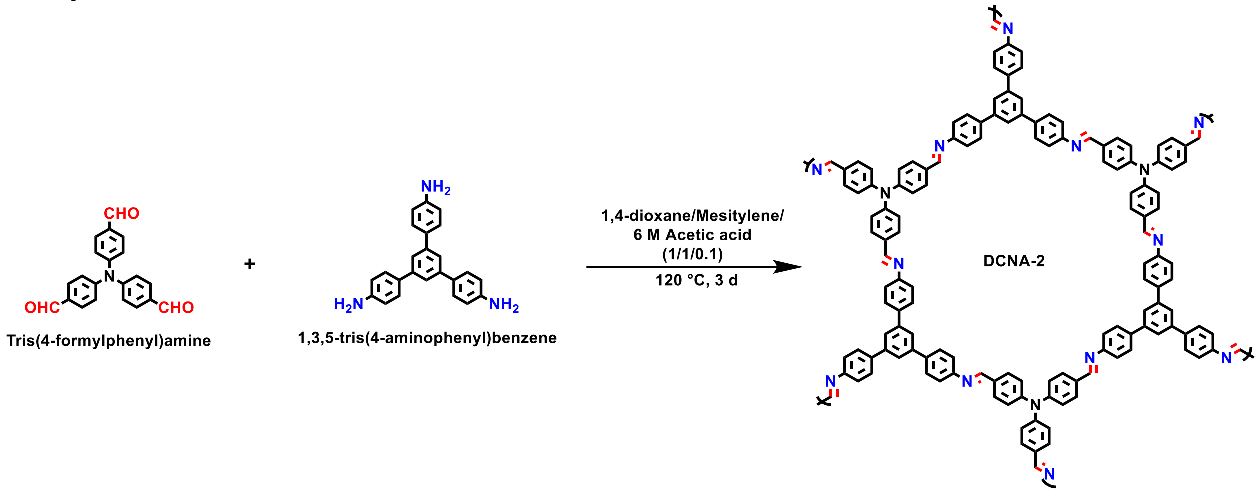

A Pyrex glass tube (15 mL) was charged with Tris(4-formylphenyl)amine (Donor) (65.9 mg, 0.2 mmol), 1,3,5-Tris(4-aminophenyl)benzene (Acceptor) (70.3 mg, 0.2 mmol), 3 mL dioxane, 3 mL mesitylene and 0.3 mL 6 M acetic acid aqueous solution. The tube was first sonicated until a fluffy solid formed and then flash frozen at 77 K (liquid N_2 bath) and degassed by three times freeze-pump-thaw cycles. The internal pressure was evacuated to 10^{-3} mbar. The tube was sealed and placed in a preheated oven at 120 °C for 3 days. After finishing heating, the tube was cooled down and cut. The formed yellow precipitate was filtered and washed with acetone several times. Finally, the DCNA-2 powder was dried in a normal oven at 80 °C. Yield = 94.1% (118 mg)

2.4 Synthesis of DNCA-2:

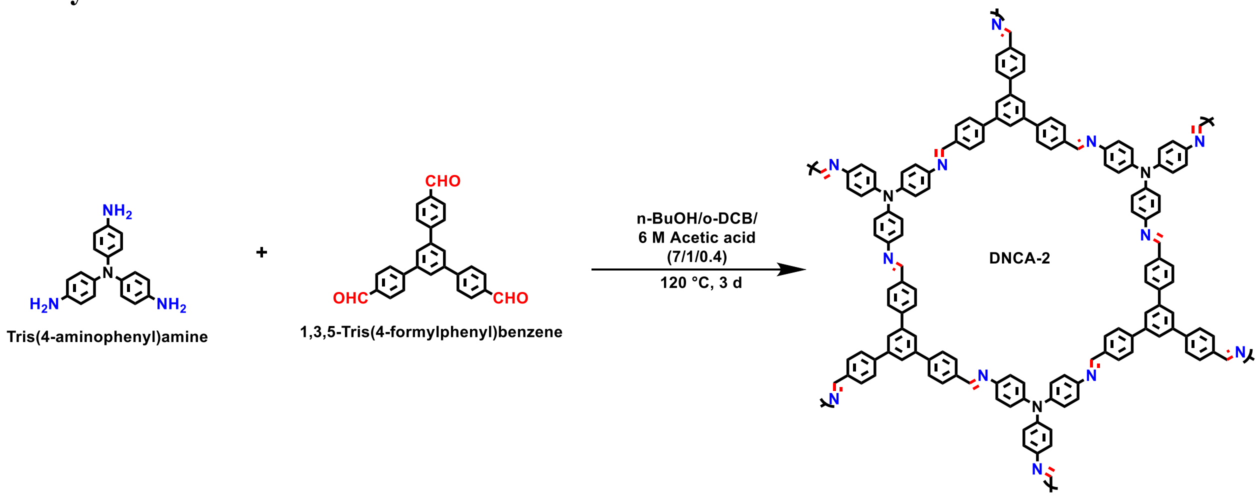

A Pyrex glass tube (15 mL) was charged with Tris(4-aminophenyl)amine (Donor) (43.5 mg, 0.15 mmol), 1,3,5-Tris(4-formylphenyl)benzene (Acceptor) (58.6 mg, 0.15 mmol), 5.25 mL n-BuOH, 0.75 mL o-DCB and 0.3 mL 6 M acetic acid aqueous solution. The tube was first sonicated until a fluffy solid formed and then flash frozen at 77 K (liquid N_2 bath) and degassed by three times freeze-pump-thaw cycles. The internal pressure was evacuated to 10^{-3} mbar. The tube was sealed and placed in a preheated oven at 120 °C for 3 days. After finishing heating, the tube was cooled down and cut. The formed brown-orange precipitate was filtered and washed with acetone several times. Finally, the DNCA-2 powder was dried in a normal oven at 80 °C. Yield = 89.4 % (84 mg)

2.5 Synthesis of DCNA-3[17]:

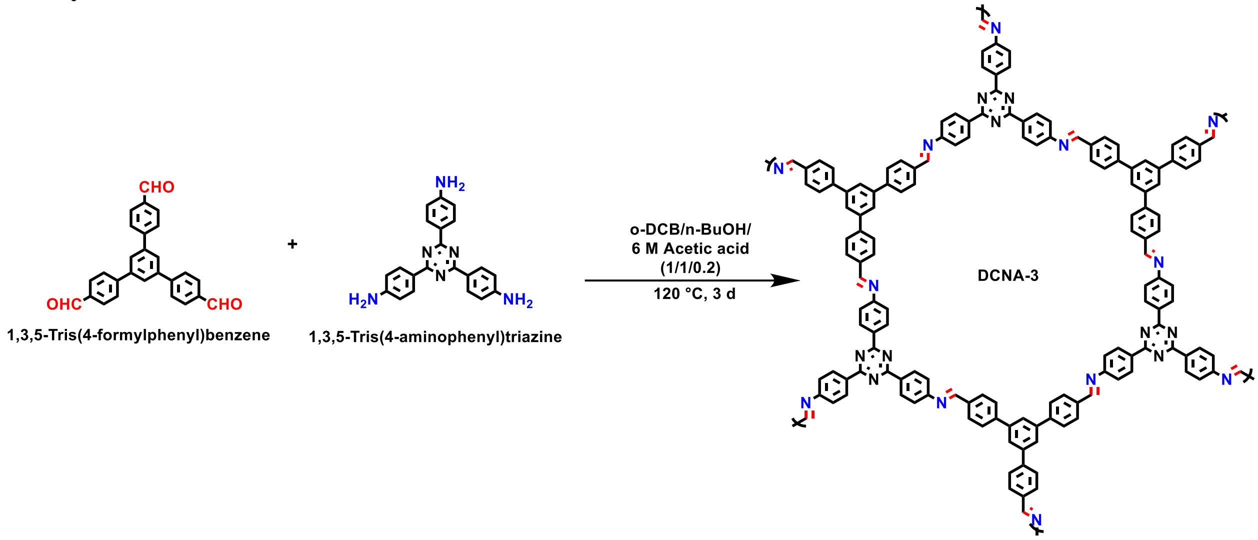

For the synthesis of DCNA-3, a Pyrex glass tube (15 mL) was charged with 1,3,5-Tris(4-formylphenyl)benzene (Donor) (58.6 mg, 0.15 mmol), 1,3,5-Tris(4-aminophenyl)triazine (Acceptor) (53.2 mg, 0.15 mmol), 2 mL o-DCB, 2 mL n-BuOH and 0.4 mL 6 M acetic acid aqueous solution. The tube was first sonicated for 30 minutes to form bulk solid and then flash frozen at 77 K (liquid N_2 bath) and degassed by three times freeze-pump-thaw cycles. The internal pressure was evacuated to 10^{-3} mbar. The tube was sealed and heated at 120 °C for 3 days. The green-yellow precipitate was washed with acetone several times and collected by filtration. Finally, the powder was dried in a normal oven at 80 °C. Yield = 89.7 % (93 mg).

2.6 Synthesis of DNCA-3:

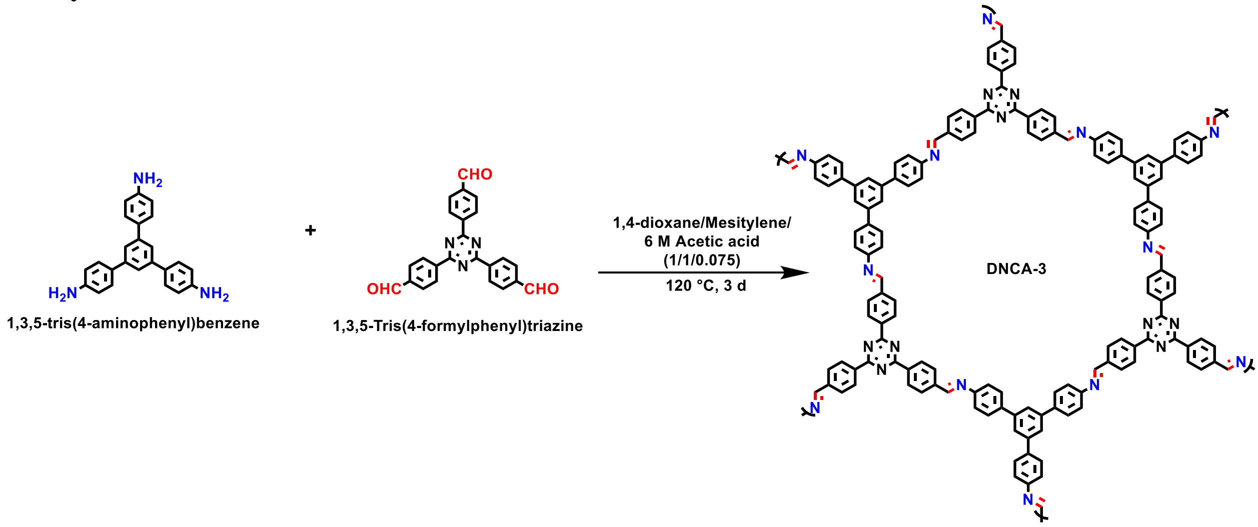

For the synthesis of DNCA-3, a Pyrex glass tube (15 mL) was charged with 1,3,5-Tris(4-aminophenyl)benzene (Donor) (35.2 mg, 0.1 mmol), 1,3,5-Tris(4-formylphenyl)triazine (Tta) (39.3 mg, 0.1 mmol), 2 mL 1,4-dioxane, 2 mL mesitylene and 0.15 mL 6 M acetic acid aqueous solution. The tube was first sonicated until forming fluffy solid and then flash frozen at 77 K (liquid N_2 bath) and degassed by three times freeze-pump-thaw cycles. The internal pressure was evacuated to 10^{-3} mbar. The tube was sealed and heated at 120 °C for 3 days. The yellow precipitate was washed with acetone several times and collected by filtration. Finally, the powder was dried in a normal oven at 80 °C. Yield = 98.8 % (68.2 mg).

2.7 Synthesis of the isomeric protonated COFs:

Typically, 30 mg of the pristine COF was stirred in 16 mL 0.1 mol L^{-1} (M) AC aqueous solution for 5 minutes following by filtration and drying under ultra-high vacuum at room temperature overnight to obtain AC modified COFs.

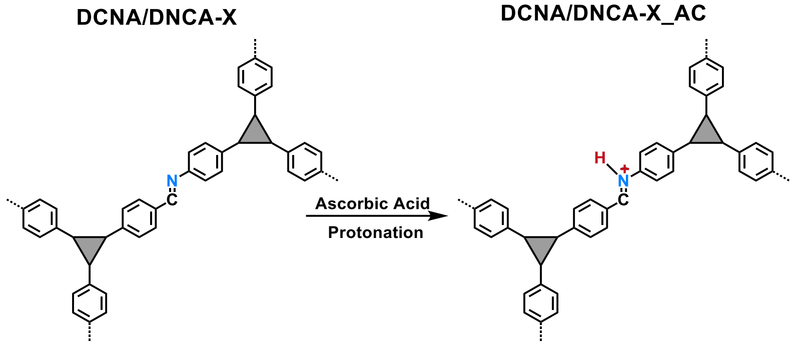

Supplementary Note 3. Characterization of the isomeric COFs

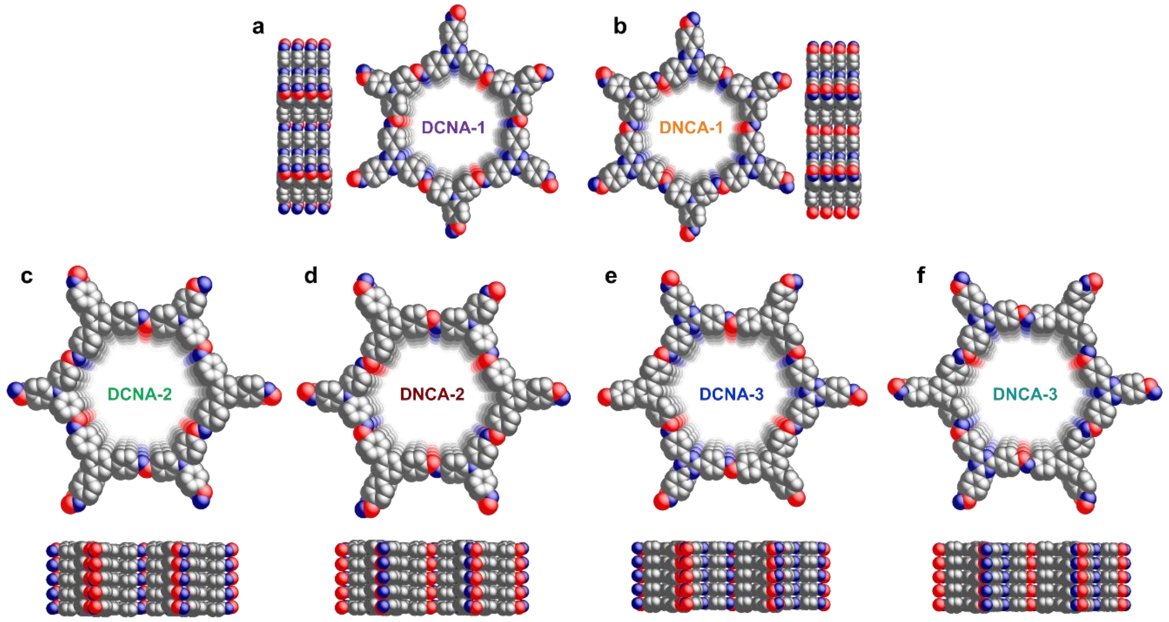

Supplementary Figure 1. Crystal structures of the isomeric COFs. DCNA-1 (a), DNCA-1 (b), DCNA-2 (c), DNCA-2 (d), DCNA-3 (e), DNCA-3 (f).

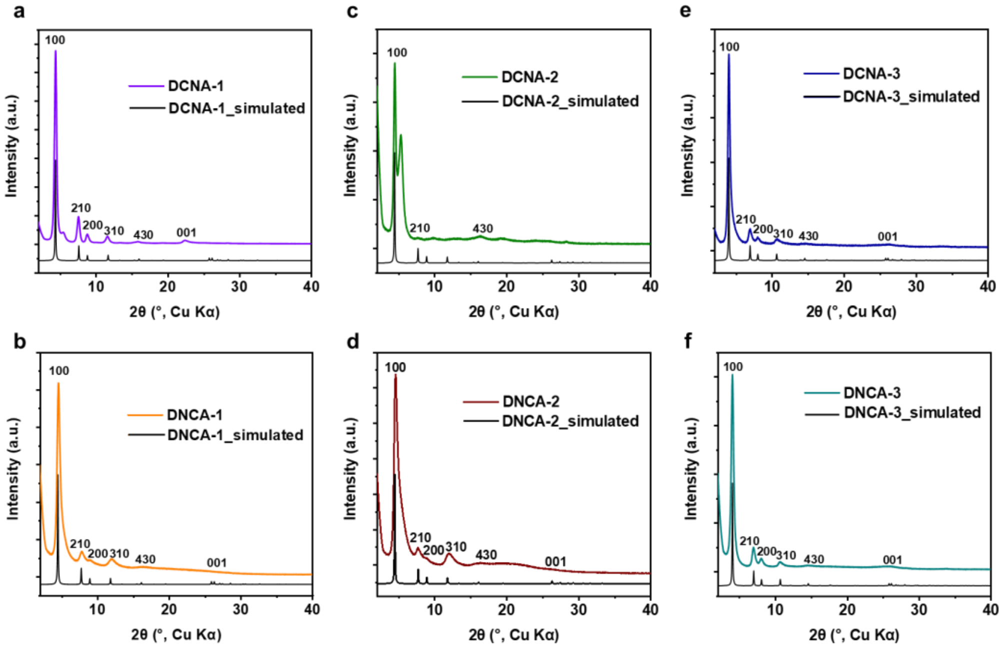

Supplementary Figure 2. Experimental and simulated PXRD patterns of the isomeric COFs. DCNA-1 (a), DNCA-1 (b), DCNA-2 (c), DNCA-2 (d), DCNA-3 (e), DNCA-3 (f). (The diffraction peaks at about 5°, mainly visible for DCNA-2, can be probably attributed to a pronounced disorder in the stacking direction of the COF [19,20])

The crystallinity of the synthesized COFs was evaluated by powder X-ray diffraction (PXRD, Cu Kα radiation, λ = 1.5418 Å) analyses (Supplementary Figure 2). Generally, all the synthesized COFs showed sharp diffraction peaks. The first intense reflection observed in the experimental patterns can be assigned to the (100) facets of a primitive hexagonal lattice, and the broad diffraction peaks at around 25° can be assigned to the (001) facets of the π-π stacking. The crystal structure of most of these isomeric COFs has been refined previously,[17] a similar procedure was used for the COF not described in the literature. For all the COFs, AA and AB stacking models were constructed, and their corresponding PXRD patterns were calculated and

compared to the experimental PXRD patterns Furthermore, the experimental PXRD patterns. A better match was systematically observed with the calculated patterns of the fully eclipsed (AA) models (Supplementary Figure 1).
The diffraction peak at about 5º of DCNA-1 and DNCA-1, not found in the simulated patterns, has been also observed in other 2D COFs and was attributed to a pronounced disorder in the stacking direction.[19,20] Despite several attempts, we could not satisfactorily identify a reasonable representation of this disordered stacking patterns.

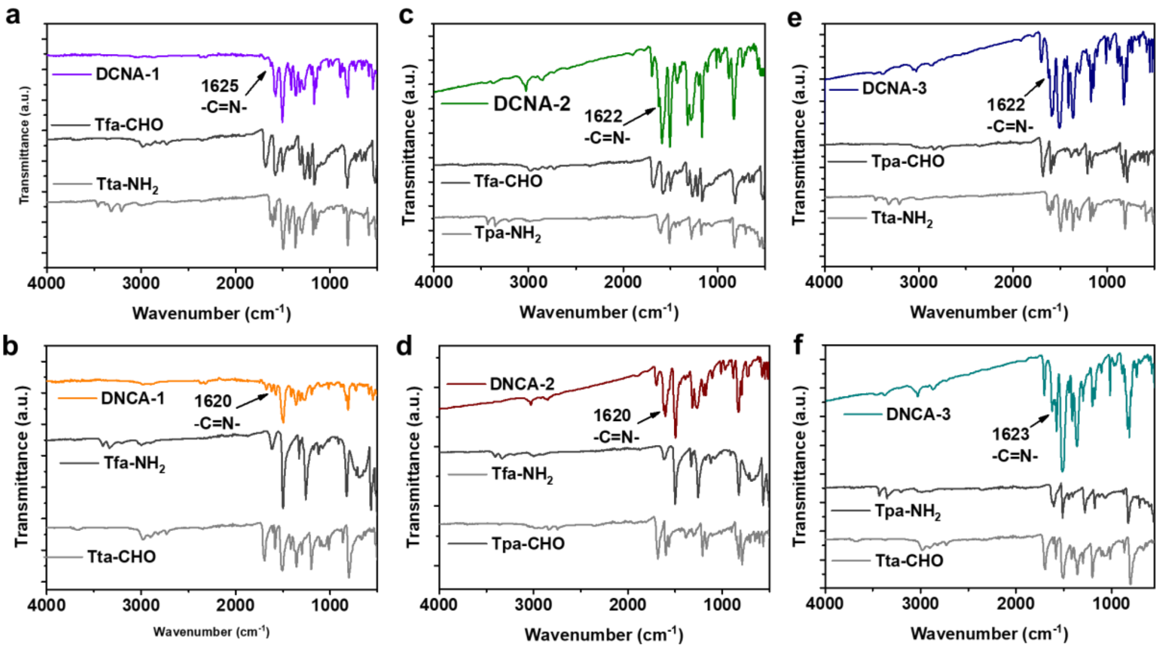

Supplementary Figure 3. FTIR spectra of the isomeric COFs. DCNA-1 (a), DNCA-1 (b), DCNA-2 (c), DNCA-2 (d), DCNA-3 (e), DNCA-3 (f) and FTIR of the corresponding starting materials.

FTIR measurements confirmed the chemical structure of the isomeric COFs. All the experimental spectra of the COFs exhibit the almost disappearance of the stretching bands of the starting aldehyde and amine monomers, and the appearance of a prominent imine vibration peak at around 1620 cm^{-1}, indicating the complete condensation of the monomers and the successful formation of the imine bond (Supplementary Figure 3). The different wavenumbers of the -C=N- stretching in DCNA and DNCA COFs indicate the different chemical environments of -C=N-, proving their different directions.

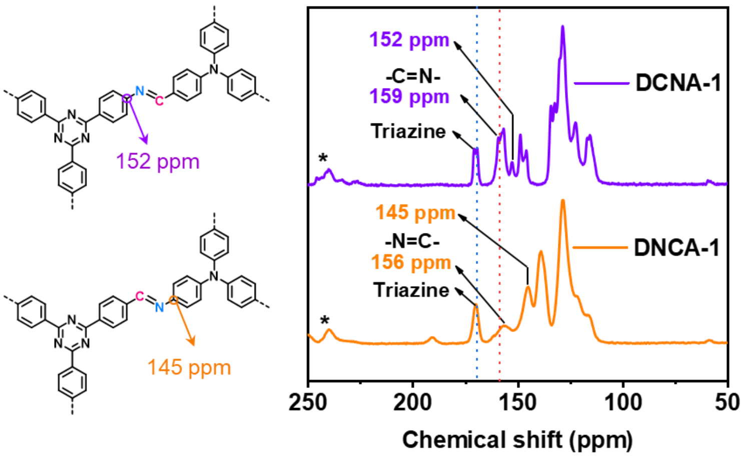

Supplementary Figure 4. ^{13}C CP-MAS solid-state NMR spectra (* spinning sidebands) of the isomeric COFs DCNA-1 and DNCA-1.

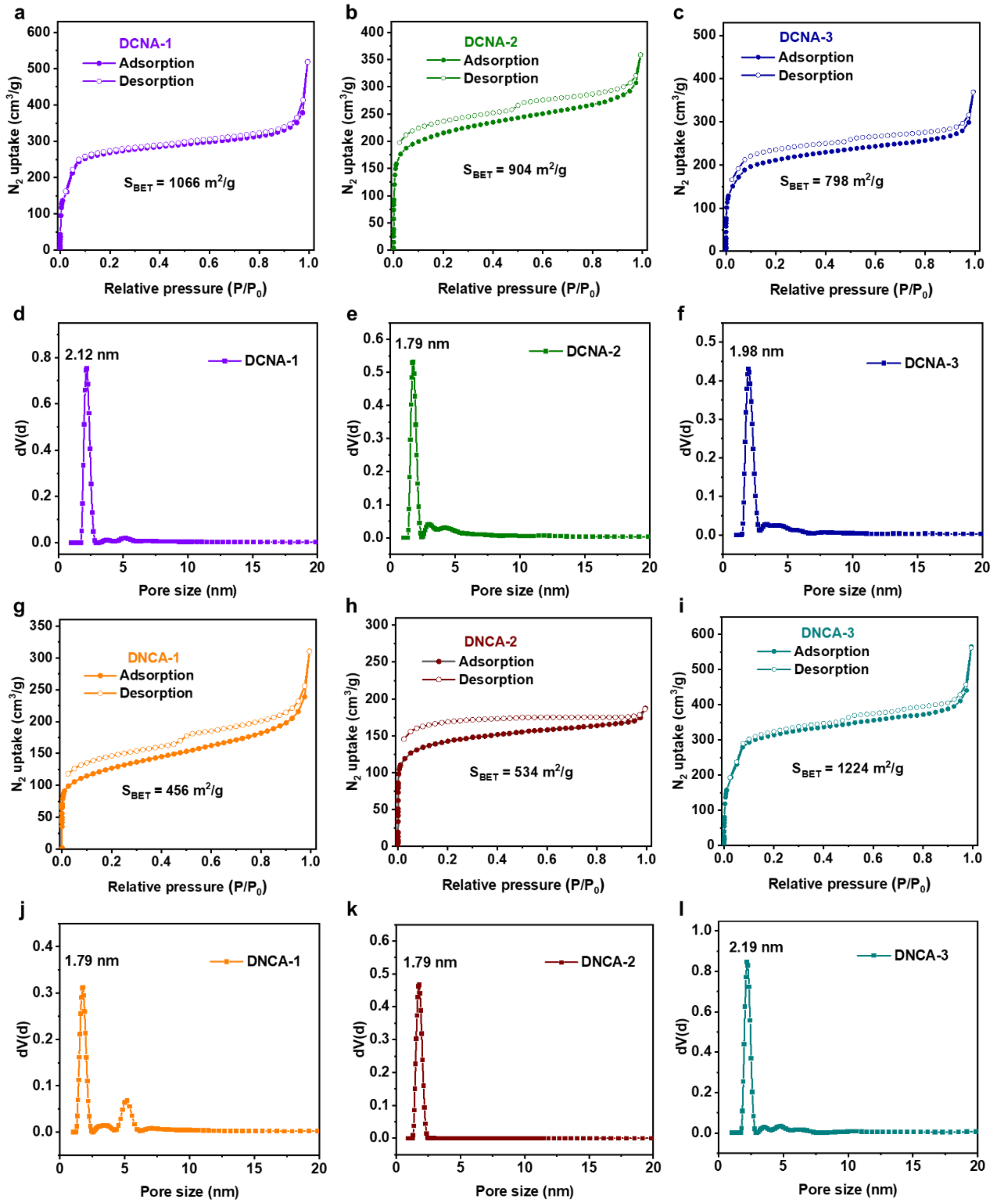

Supplementary Figure 5. N_2 isotherms (at 77K) of the isomeric COFs. DCNA-1 (a), DCNA-2 (b), DCNA-3 (c) and DNCA-1 (g), DNCA-2 (h), DNCA-3 (i). Calculated pore size distribution plot of DCNA-1 (d), DCNA-2 (e), DCNA-3 (f) and DNCA-1 (j), DNCA-2 (k), DNCA-3 (l) from N_2 adsorption branches of N_2 isotherms.

N_2 sorption was applied to evaluate the porosity of the synthesized COFs. In general, all the COFs show high porosity and narrow pore size distribution, which attributes to the ordered cylinder pores in the crystal structure. The Brunauer–Emmett–Teller (BET) surface area of DCNA-1, DCNA-2, DCNA-3 was calculated to be 1066, 904, and 798 m^2 g^{-1}, respectively (Supplementary Figures 5a-c). The experimental pore size distribution derived from the adsorption data was determined to be centered at 2.12, 1.79, and 1.98 nm for DCNA-1, DCNA-2, DCNA-3, respectively (Supplementary Figures 5d-f). Similarly, the BET surface area of DNCA-1, DNCA-2, DNCA-3 was calculated to be 456, 534, and 1224 m^2 g^{-1}, respectively (Supplementary Figures 5g-i). The experimental pore size distribution derived from the adsorption data was determined to be centered at 1.79, 1.79, and 2.19 nm, respectively (Supplementary Figures 5j-l).

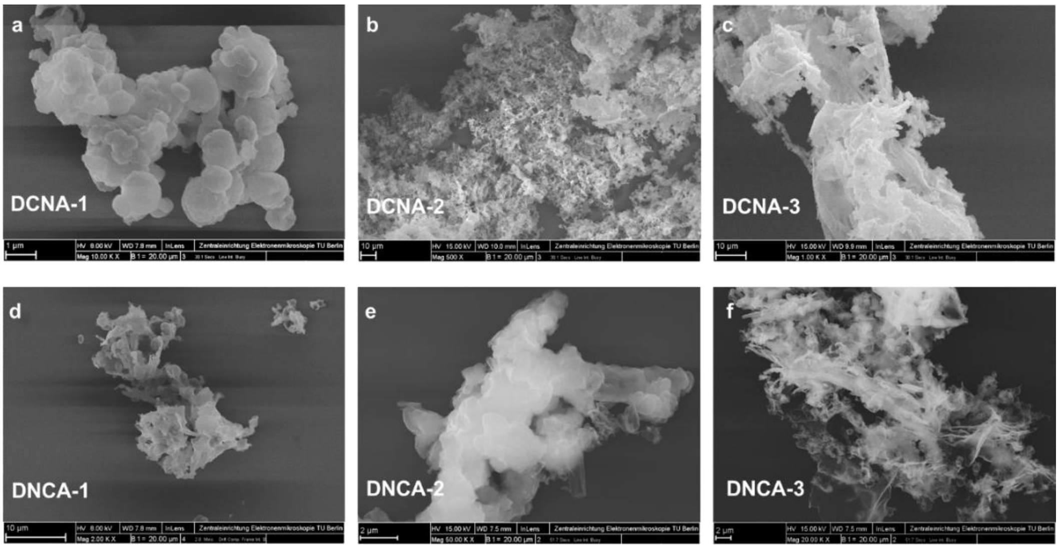

Supplementary Figure 6. SEM images of the isomeric COFs. DCNA-1 (a), DCNA-2 (b), DCNA-3 (c), DNCA-1 (d), DNCA-2 (e), DNCA-3 (f).

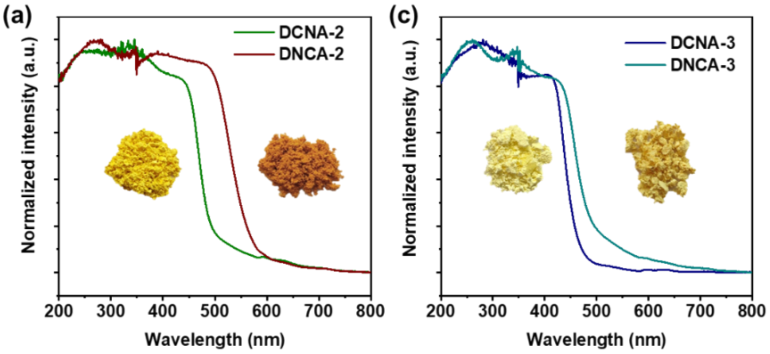

Supplementary Figure 7. UV-Vis DRS spectra of DCNA-2 and DNCA-2 (a), DCNA-3, and DNCA-3 (b). Insets are the photographs of the COFs.

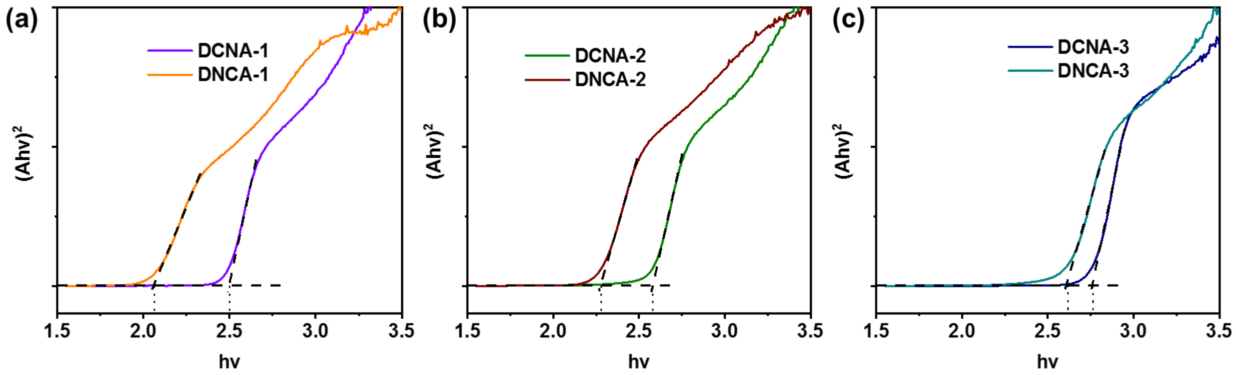

Supplementary Figure 8. Bandgap's calculation of the DCNA and DNCA COFs.

UV-Vis DRS spectra illustrate the light absorption abilities of the COFs. All DNCA COFs show red-shifted light absorption than all DCNA COFs (Fig. 2e, Supplementary Figure 7). The absorption onsets of pristine COFs DCNA-1, DCNA-2, and DCNA-3 are located at 520, 497, and 465 nm, respectively, which red-shifted to 645, 570, and 497 nm for pristine COFs DNCA-1, DNCA-2, and DNCA-3, respectively. Accordingly, the band gaps calculated from Tauc plots are 2.48, 2.57, and 2.76 eV for DCNA-1, DCNA-2, and DCNA-3. And the bandgaps of DNCA-1, DNCA-2, and DNCA-3 are calculated to be 2.06, 2.26, and 2.61 eV, respectively (Supplementary Figure 8).

Supplementary Note 4. Characterization of the protonated COFs

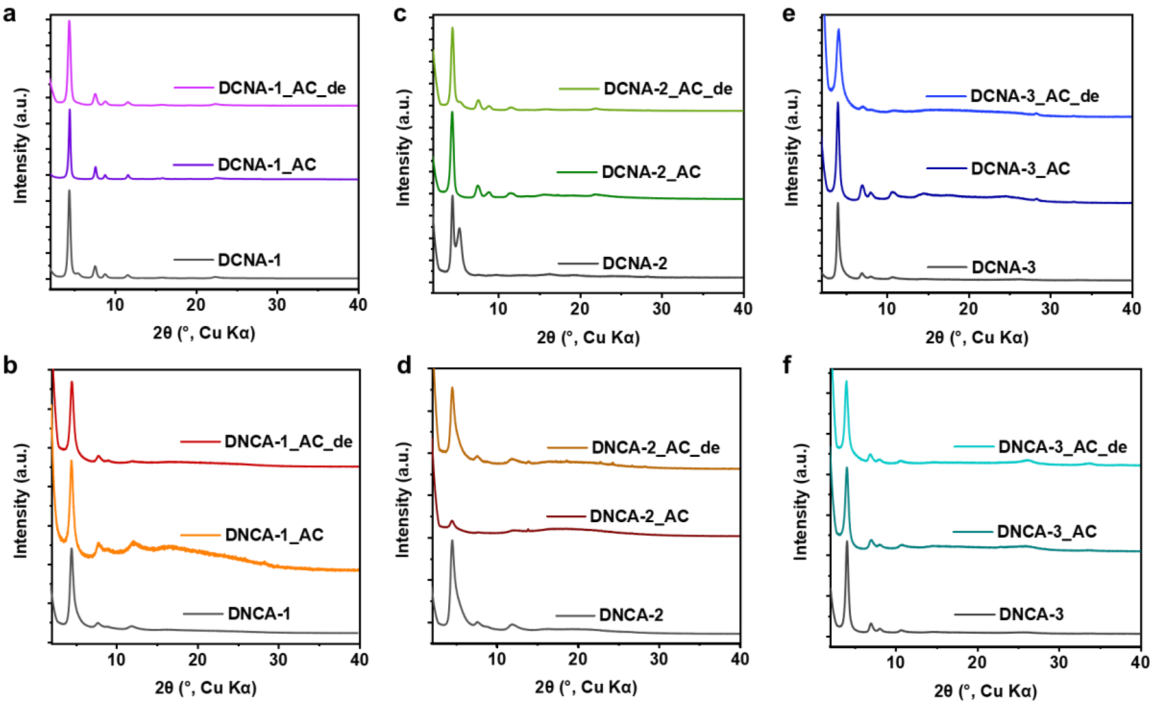

Supplementary Figure 9. Comparison of the PXRD patterns of the isomeric pristine, protonated, and deprotonated COFs. (a) DCNA-1/DCNA-1_AC/DCNA-1_AC_de, (b) DNCA-1/DNCA-1_AC/DNCA-1_AC_de, (c) DCNA-2/DCNA-2_AC/DCNA-2_AC_de, (d) DNCA-2/DNCA-2_AC/DNCA-2_AC_de (e) DCNA-3/DCNA-3_AC/DCNA-3_AC_de, (f) DNCA-3/DNCA-3_AC/DNCA-3_AC_de. "_AC" stands for AC protonation. "_de" stands for deprotonation of the protonated COFs. The preparation of the protonated samples is described in Supplementary Note 2.7. The deprotonation process was performed by washing the protonated COFs with water and acetone thoroughly then drying under vacuum at room temperature. The color of the COFs reverted to the color of the pristine COFs.

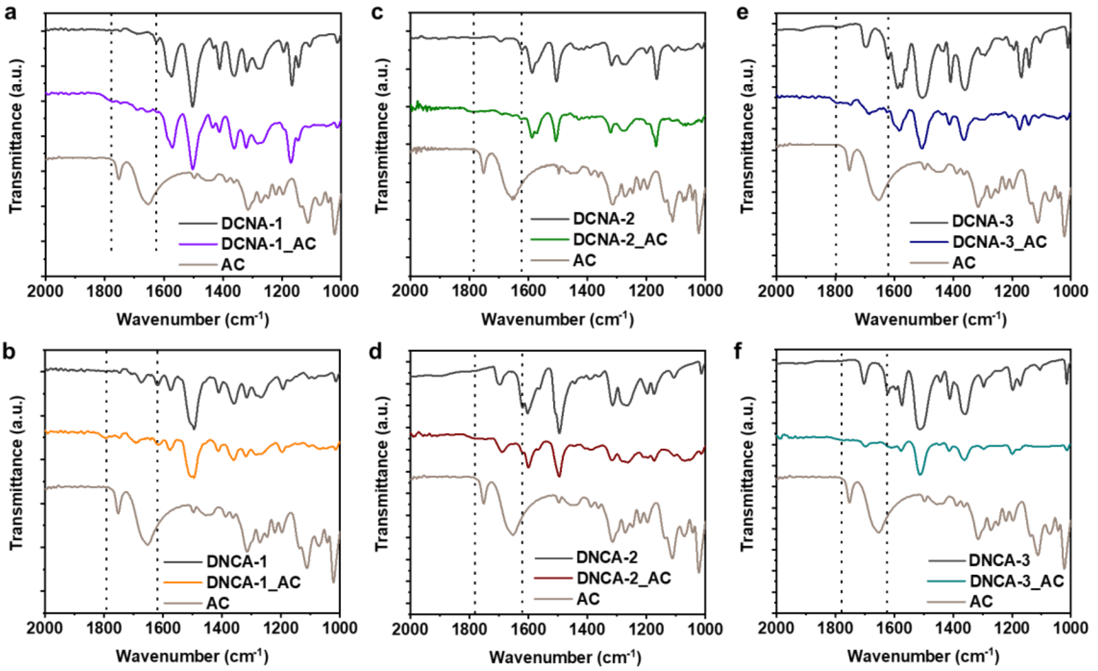

Supplementary Figure 10. Comparison of the FTIR spectra of isomeric pristine COFs, protonated COFs, and AC. DCNA-1/DCNA-1_AC (a), DNCA-1/DNCA-1_AC (b), DCNA-2/DCNA-2_AC (c), DNCA-2/DNCA-2_AC (d), DCNA-3/DCNA-3_AC (e), DNCA-3/DNCA-3_AC (f).

In the FTIR spectra of DCNA_AC and DNCA_AC, new peaks appeared at around 1790 cm^{-1} (broad), which cannot be attributed to the free AC but the newly formed $C=NH^+$ bond. Moreover, the peaks from the stretching mode of imine bonds at around 1620-1625 cm^{-1} disappeared or attenuated (Supplementary Figure 10), confirming the protonation of the imines[17].

Supplementary Table 1. Quantitative analysis of the protonation process via X-ray photoelectron spectroscopy (XPS)

COFs	N content (atomic%)	Imine content (mol%)	O content (atomic%)	AC content (mol%)	Protonated Imine content (mol%)
DCNA-1_AC	7.97	3.42	11.36	1.89	55.36
DNCA-1_AC	10.42	4.47	8.92	1.49	33.33
DCNA-2_AC	4.6	3.45	14.28	2.38	68.99
DNCA-2_AC	6.58	4.94	21.91	3.65	73.89
DCNA-3_AC	7.99	4.00	8.53	1.42	35.50
DNCA-3_AC	8.66	4.33	7.84	1.31	30.25

The protonation degree is calculated via the equation. Protonated Imine content = $\frac{\text{AC content}}{\text{Imine content}}$

Note that DCNA-1_AC has a significantly higher protonation degree than DNCA-1_AC, which should additionally influence its photocatalytic performance. On the other hand, DCNA-2_AC and DCNA-3_AC show similar protonation degrees as their isomers. Furthermore, protonation degrees are higher for the DCNA/DNCA-2 pair than for DCNA/DNCA-3 (Supplementary Table 1). As the trend of band structure change and photocatalytic HER activity is however the same for all three protonated COF pairs, it can be concluded that the protonation degree in all COFs is high enough to substantially change the intrinsic properties of all isomeric COFs and that thus the orientation of the linkage is the most decisive factor (Supplementary Tables 3 and 4).

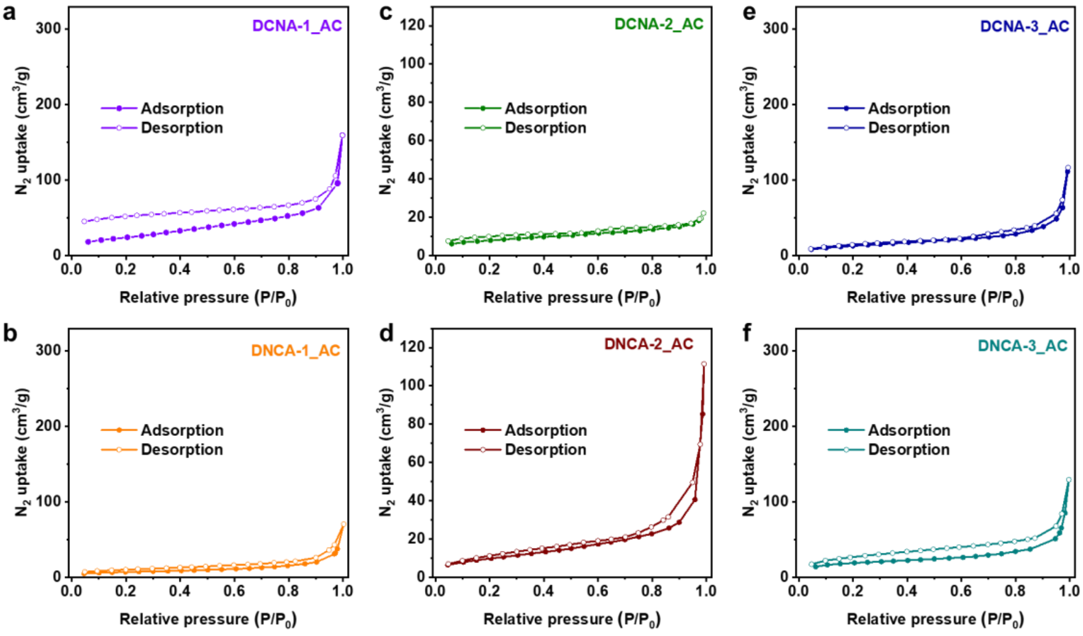

Supplementary Figure 11. N_2 isotherms (at 77K) of the isomeric protonated COFs. DCNA-1_AC (a), DNCA-1_AC (b), DCNA-2_AC (c), DNCA-2_AC (d), DCNA-3_AC (e), DNCA-3_AC (f).

Supplementary Note 5. Comparison of pristine and protonated COFs

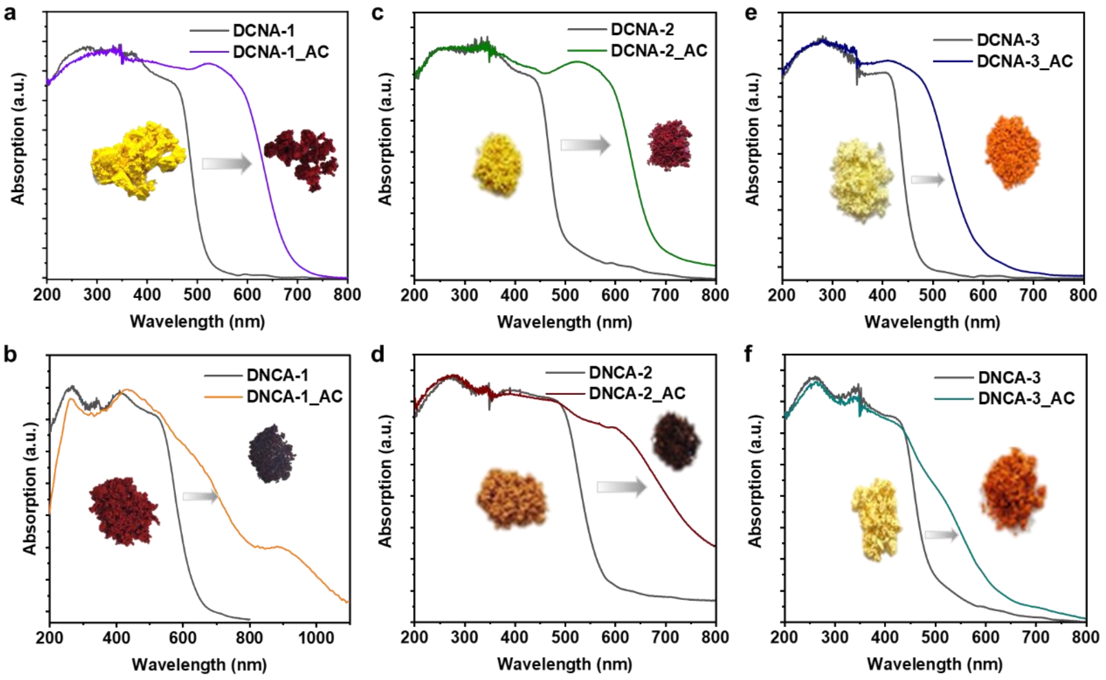

Supplementary Figure 12. Comparison of the UV-Vis spectra of isomeric pristine and protonated COFs. DCNA-1/DCNA-1_AC (a), DNCA-1/DNCA-1_AC (b), DCNA-2/DCNA-2_AC (c), DNCA-2/DNCA-2_AC (d), DCNA-3/DCNA-3_AC (e), DNCA-3/DNCA-3_AC (f).

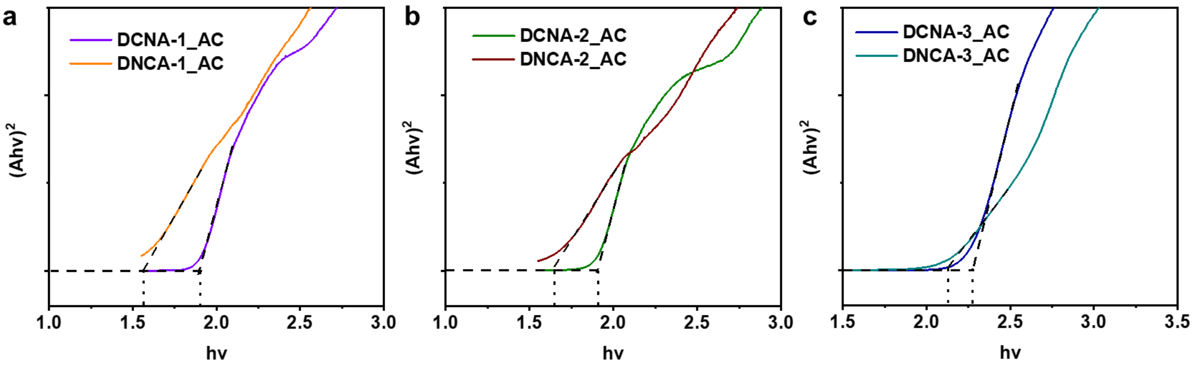

Supplementary Figure 13. Bandgaps calculation of the protonated COFs.

After protonation, remarkably darkened color change has been observed for all the COFs (insets of Supplementary Figure 12). The absorption onsets of protonated COFs DCNA-1_AC, DCNA-2_AC, and DCNA-3_AC are located at 685, 689, and 593 nm, respectively, which red-shifted as well to 916, 874, and 639 nm for protonated COFs DNCA-1_AC, DNCA-2_AC, and DNCA-3_AC, respectively. The bandgaps calculated from Tauc plots are 1.90, 1.90, and 2.28 eV for DCNA-1_AC, DCNA-2_AC, and DCNA-3_AC. And the band gaps of DNCA-1_AC, DNCA-2_AC, and DNCA-3_AC are calculated to be 1.56, 1.65, and 2.12 eV, respectively (Supplementary Figure 13).

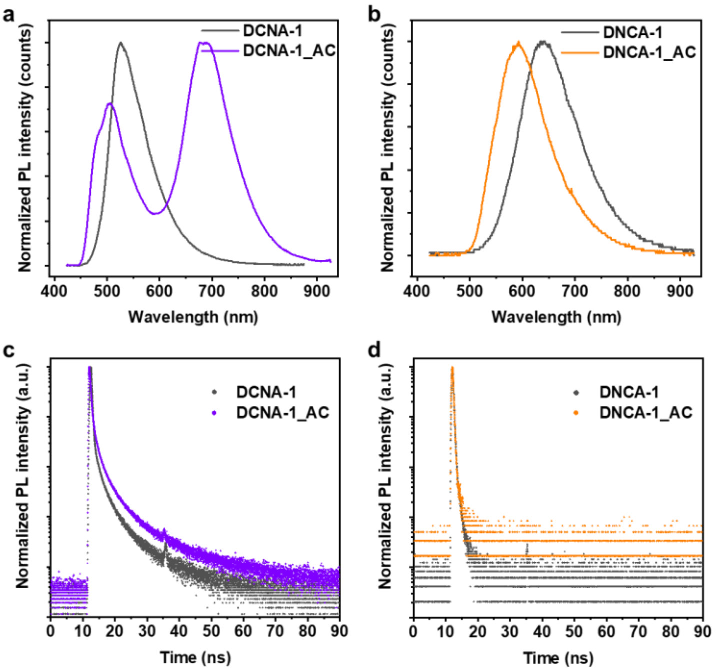

Supplementary Figure 14. Comparison of the PL spectra of DCNA-1 and DCNA-1_AC (a), DNCA-1, and DNCA-1_AC (b). Comparison of the PL decay of DCNA-1 and DCNA-1_AC (c), DNCA-1, and DNCA-1_AC (d).

The PL spectra of DCNA-1 and DNCA-1 were measured in solid-state (Supplementary Figure 14). Upon excitation at 405 nm, the emission maximum of DCNA-1 appeared at 526 nm. Meanwhile, for DNCA-1, this maximum was distinctly red-shifted to 639 nm. After protonation, both DCNA and DNCA COFs exhibited unusual photophysical properties. DCNA-1_AC showed two peaks in the PL spectrum. There is no solid explanation for the double-peak emission in the PL spectra of DCNA-1_AC. The reason might be an excited-state intramolecular proton-transfer (ESIPT) phenomenon as the protonation introduced proton donor –OH (in AC), which can interact with the proton acceptor –C=N–[23]. For DNCA-1_AC a red-shift in absorption compared to DNCA-1 was observed (see supp. Fig. 12) but a blue-shift in photoluminescence.

Supplementary Table 2. The fitted PL decays results of DCNA-1_AC and DNCA-1_AC with three component exponential decay functions.

	τ_3 (ns)	A_3	τ_2 (ns)	A_2	τ_1 (ns)	A_1	T_{avg} (ns)
DCNA-1_AC	16.5	1.2×10^{-3}	3.45	0.032	0.510	1.0	0.620
DNCA-1_AC	0.315	1.1	2.05	0.0050	-	-	0.323

The PL decays were recorded, and the average PL lifetimes were calculated via fitting the decays with three component exponential decay functions. DCNA-1 and DNCA-1 PL showed a lifetime of 0.367 ns and 0.325 ns, respectively (Supplementary Figure 14, Table 2). Thus, a longer lifetime of the excited state of DCNA-1 was observed. The protonated COFs DCNA-1_AC and DNCA-1_AC showed a PL lifetime of 0.620 ns and 0.323 ns, respectively. There were no observable differences between the PL decay of DNCA-1 and DNCA-1_AC. The PL decay of DNCA-1 and DNCA-1_AC was faster than DCNA-1 and DCNA-1_AC. Considering the fast PL decay in DNCA-1 and DNCA-1_AC, it can be inferred that the non-radiative decay of excitons/charge carriers was the dominant decay channel in these COFs. However, further evidence on the defects/disorders in DNCA-1/DNCA-1_AC compared to DCNA-1/DCNA-1_AC is needed.

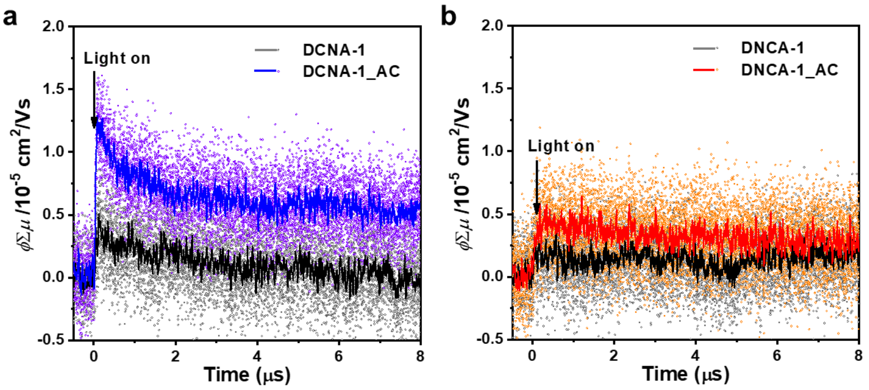

Supplementary Figure 15. Comparison of the FP -TRMC spectra of DCNA-1 and DCNA-1_AC (a), DNCA-1, and DNCA-1_AC (b).

Generally, both the protonated forms of the COFs – DCNA-1_AC and DNCA-1_AC showed enhanced photoconductivity, evidenced by the enhanced FP-TRMC signal intensity. Specifically, a significant three-fold enhancement in photoconductivity was observed for DCNA-1 after protonation, whereas a slight enhancement was observed for DNCA-1 after the protonation.

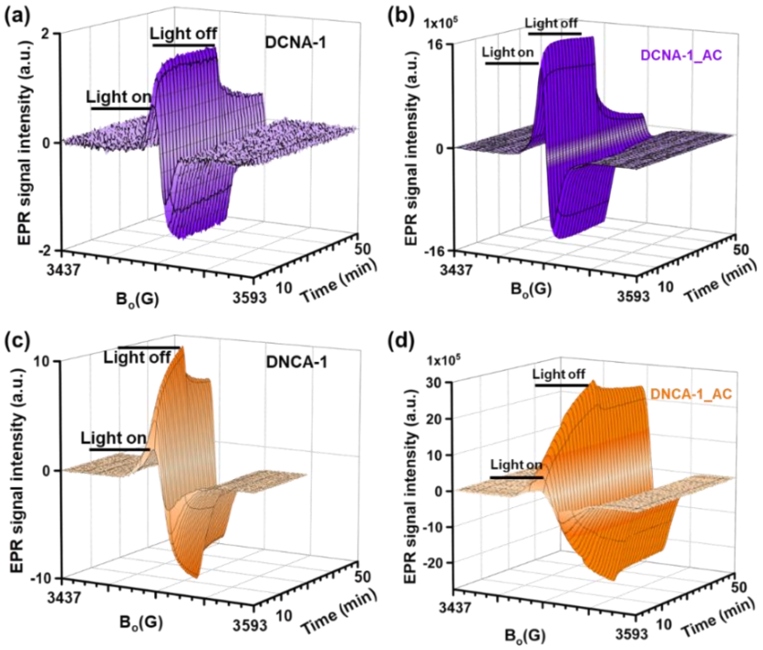

Supplementary Figure 16. Comparison of the EPR CB electrons spectra of the isomeric COFs before and after protonation. DCNA-1 (a), DCAN-1_AC (b), DNCA-1 (c), and DNCA-1_AC (d). (Light source: >420 nm, 300 W Xe lamp).

Both the protonated forms of the COFs, DCNA-1_AC and DNCA-1_AC show remarkably enhanced EPR CB electron signal than their pristine COFs, which proves that protonation facilitates the charge separation efficiency. This trend is also identical to our previous report[17]. Furthermore, protonation did not change the CB electron intrinsic behaviors of DCNA-1_AC and DNCA-1_AC comparing with their pristine forms. That is, DCNA-1_AC responds faster to light irradiation, but its charge separation efficiency (indicated by the signal intensity) is lower than DNCA-1_AC (Supplementary Figure 16).

Supplementary Note 6. Photocatalytic H_2 evolution

The photocatalysis was conducted in a 36 mL side irradiation quartz reactor equipped with a 152 mL glass gas container. Generally, 3 mg pristine COF is dispersed in either 16 mL 0.1 M AC aqueous or triethanolamine (TEOA)/water (v:v=2 mL: 16 mL) solution. 3 microliter H_2PtCl_6 aqueous solution (8 wt%) is added as the source of Pt co-catalyst. The reactor is sealed with rubber stoppers and degasses by Argon for 30 minutes before irradiation. Then, the reactor is irradiated with a 300 W Xe lamp (L.O.T-Quantum design) with appropriate filters. The light to reactor distance is controlled to be 10 cm. The temperature of the system is kept at 20 °C by water circulating. The gas sample is taken from the headspace, and H_2 is quantified by GC (Agilent 7820A) equipped with a thermal conductivity detector. The photocatalytic hydrogen evolution reaction (HER) rates in AC were determined from the linear regression fit of the H_2 evolution curves, and the pressure increase was neglected in the calculations. Due to the negligible amount of H_2 evolved by DNCA/DNCA-X_AC COFs, H_2 was only measured after finishing the irradiation. All the reacted COFs were recovered by filtration, washed with acetone, and dried at 80 °C overnight for further characterization.

The apparent quantum efficiency (AQE) is measured with the identical setup as described above but changing the filters. The diameter of the light spot is 3 cm. The intensity of $\lambda = 420$ nm light was measured ten times and averaged to be 8.79 W/m^2. The irradiation time was 6 hours. The detected amount of substance of H_2 was 3.47 μmol for DCNA-1 COF. The AQE was calculated by the equation below.

$$\mathrm{AQE}=\frac{2\mathrm{n}_{\mathrm{H}_2}}{\mathrm{n}_{\mathrm{photon}}}=\frac{2\mathrm{nN}_{\mathrm{A}}hc}{\mathrm{AIt}\lambda}\times 100\%$$

where n is the molar amount of hydrogen, N_A is the Avogadro's constant, h is the Planck constant, c is the light velocity, A is the irradiation area, I is the intensity of the light, t is the reaction time, and λ is the wavelength of the monochromatic light (420 nm).

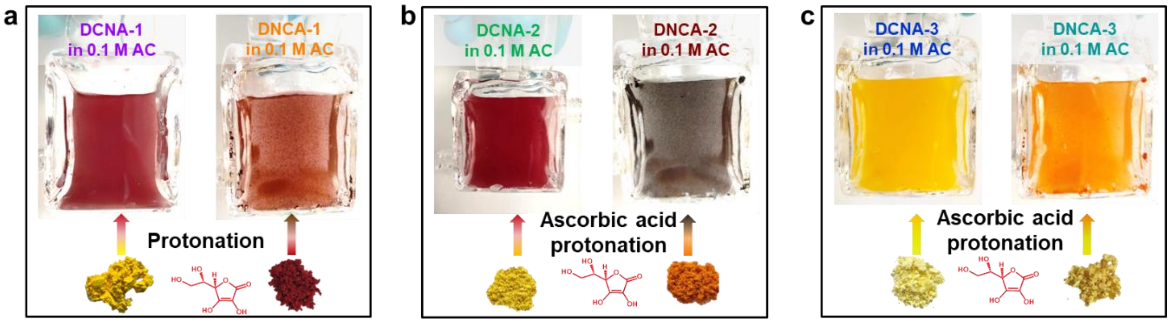

Supplementary Figure 17. The digital photographs of DCNA-1/DNCA-1 (a), DCNA-2/DNCA-2 (b), and DCNA-3/DNCA-3 (c) in 0.1 M AC reaction solution.

Supplementary Table 3. Comparison of the photocatalytic HER rate of the isomeric COFs with the unite of μmol h^{-1} and μmol g^{-1} h^{-1} in 0.1 M AC reaction solution.

COFs	HER rate in μmol h^{-1}	HER rate in μmol g^{-1} h^{-1}
DCNA-1_AC	83.66	27886.67
DNCA-1_AC	9.57	3190.00
DCNA-2_AC	58.25	19416.67
DNCA-2_AC	9.94	3313.33
DCNA-3_AC	52.81	17603.33
DNCA-3_AC	3.78	1260.00

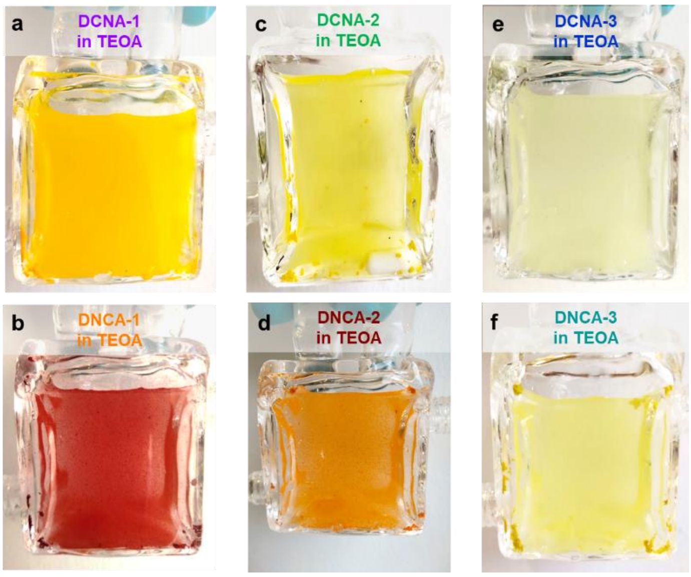

Supplementary Figure 18. The digital photographs of (a) DCNA-1, (b) DNCA-1, (c) DCNA-2, (d) DNCA-2, (e) DCNA-3, (f) DNCA-3 in TEOA aqueous solution.

The identical color of the COFs in TEOA aqueous solution to their pristine state indicated the protonation did not happen.

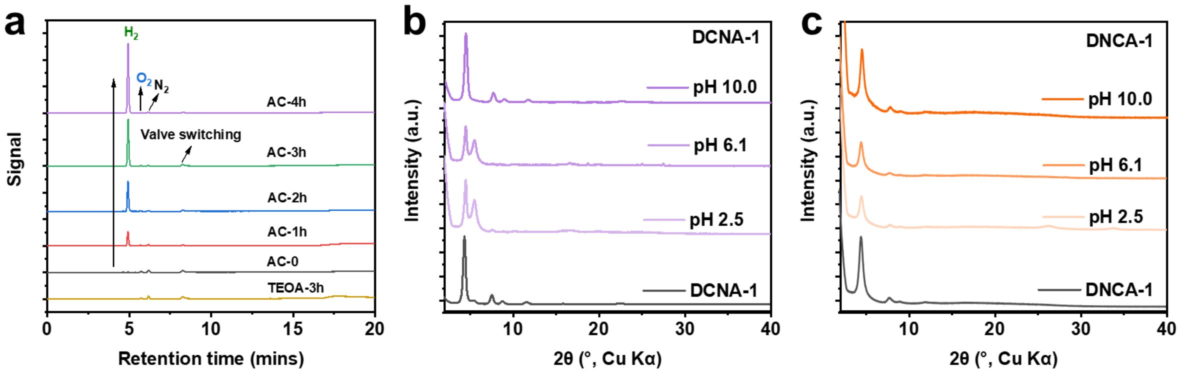

Supplementary Figure 19. (a) Representative gas chromatograph (GC) results of the photocatalysis using AC or TEOA aqueous solution as sacrificial electron donor (SED). PXRD patterns of DCNA-1 (b) and DNCA-1 (c) after 24 h treatment in different pH conditions compared with as-synthesized samples.

When the photocatalysis was conducted in 0.1 M AC, the H_2 evolved fast and continuously (Supplementary Figure 19a). In contrast, there was no obvious H_2 detected after a 3-hours reaction when TEOA was applied as the SED, verifying the negligible activity of the COFs in

TEOA (Supplementary Figure 19). In addition, there was no CO_2, and an increasing amount of N_2 detected, which excluded the possibility of COFs decomposition. And the negligible N_2 and O_2 came from the air contamination when injecting the gas samples by syringe into the GC.

The chemical stability of the COFs in different pH solutions was tested, by immersing the COFs in acidic and alkaline aqueous solutions for 24 hours, respectively. The COFs were then filtered and washed with methanol. After drying in an oven at 80°C overnight, PXRD measurements were conducted. As shown in Supplementary Figures 19 b and c, both DCNA-1 and DNCA-1 COFs retain their reflections in the PXRD after 24-hours treatment in alkaline and acidic conditions, although the lower intensity points to some loss in crystallinity.

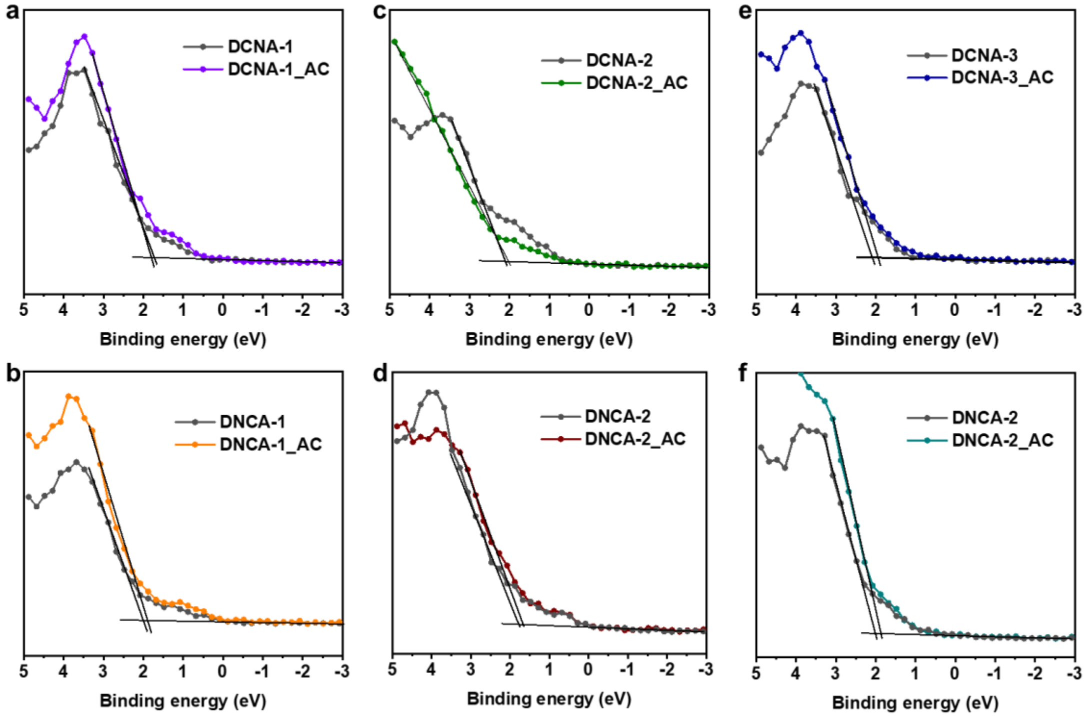

Supplementary Figure 20. VB XPS spectra of pristine and protonated COFs.

By fitting the liner parts of the onsets of the VB XPS spectra (Supplementary Figure 20), the VB-XPS values are determined and summarized in Supplementary Table 4.

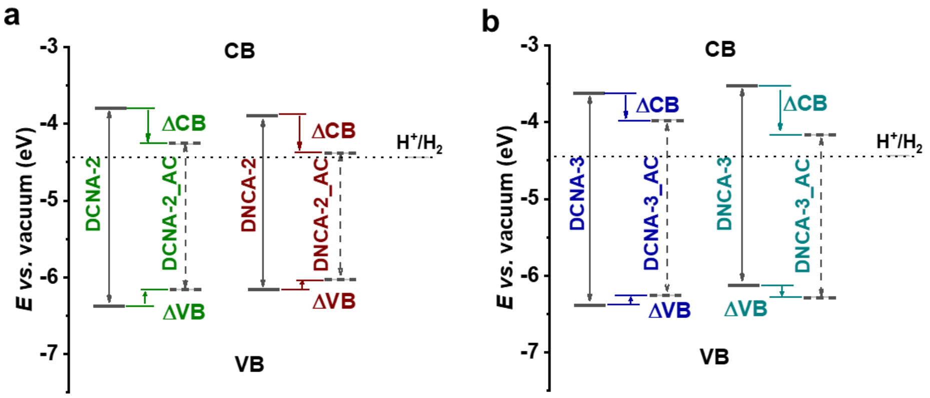

Supplementary Figure 21. The experimental band structures of the pristine and protonated COFs. (a) Comparison of DCNA-2/DCNA-2_AC and DNCA-2/DNCA-2_AC. (b) Comparison of DCNA-3/DCNA-3_AC and DNCA-3/DNCA-3_AC.

The valence band potentials (E_{VB} *vs.* vacuum level) were calculated by using eq (1), where Φ is the electron work function of the analyzer (4.35 eV)[21]. The calculated data of E_{VB} are

summarized in Supplementary Table 4.

$$E_{VB} = -(\Phi + VB_{XPS}) \quad (1)$$

The CB values were calculated according to the following equation, $Eg = | E_{VB} - E_{CB} |$.

Supplementary Table 4. Summary of the VB, CB, and bandgap values of all the studied COFs.

	Eg	$E_{(VB\text{-}XPS)}$	E_{VB} *vs.* Vacuum level	E_{CB} *vs.* Vacuum level
DCNA-1	2.48	1.69	-6.04	-3.56
DCNA-1_AC	1.90	1.75	-6.1	-4.20
DNCA-1	2.06	1.73	-6.08	-4.02
DNCA-1_AC	1.56	1.63	-5.98	-4.42
DCNA-2	2.57	2.02	-6.37	-3.80
DCNA-2_AC	1.90	1.81	-6.16	-4.26
DNCA-2	2.26	1.80	-6.15	-3.89
DNCA-2_AC	1.65	1.68	-6.03	-4.38
DCNA-3	2.76	2.03	-6.38	-3.62
DCNA-3_AC	2.28	1.90	-6.25	-3.97
DNCA-3	2.61	1.78	-6.13	-3.52
DNCA-3_AC	2.12	1.93	-6.28	-4.16

*All the values are with eV as the unit.

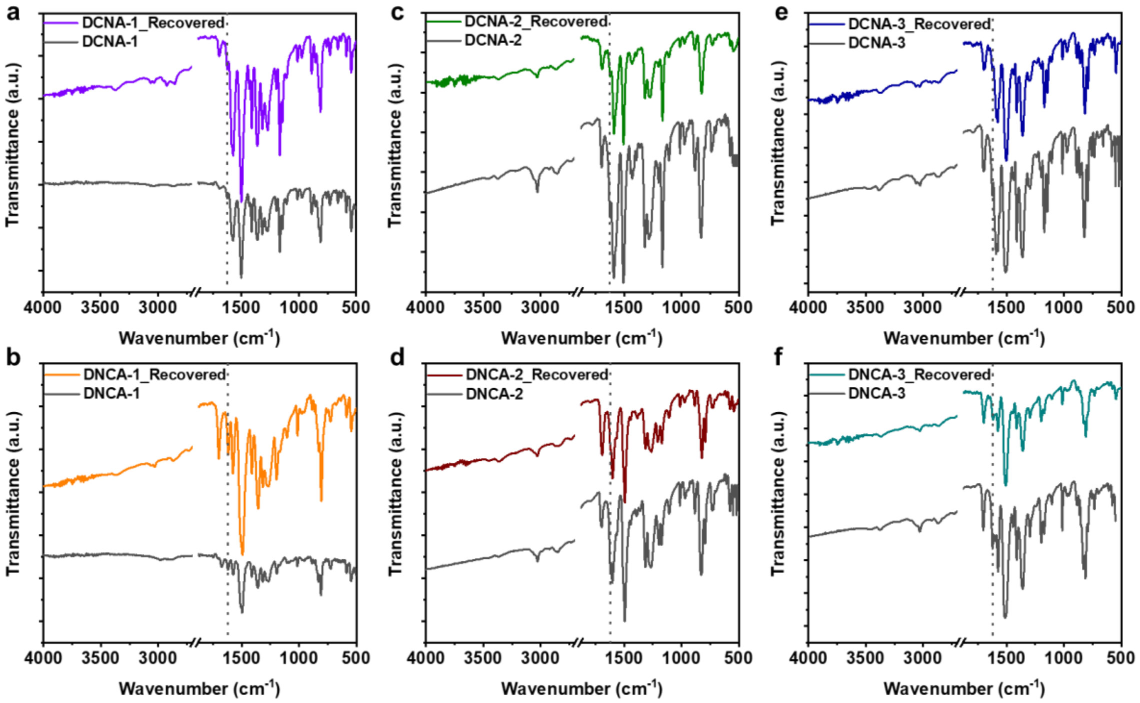

Supplementary Figure 22. FTIR spectra of the COFs recovered after photocatalysis in 0.1 M AC aqueous solution. (a) DCNA-1, (b) DNCA-1, (c) DCNA-2, (d) DNCA-2, (e) DCNA-3, (f) DNCA-3.

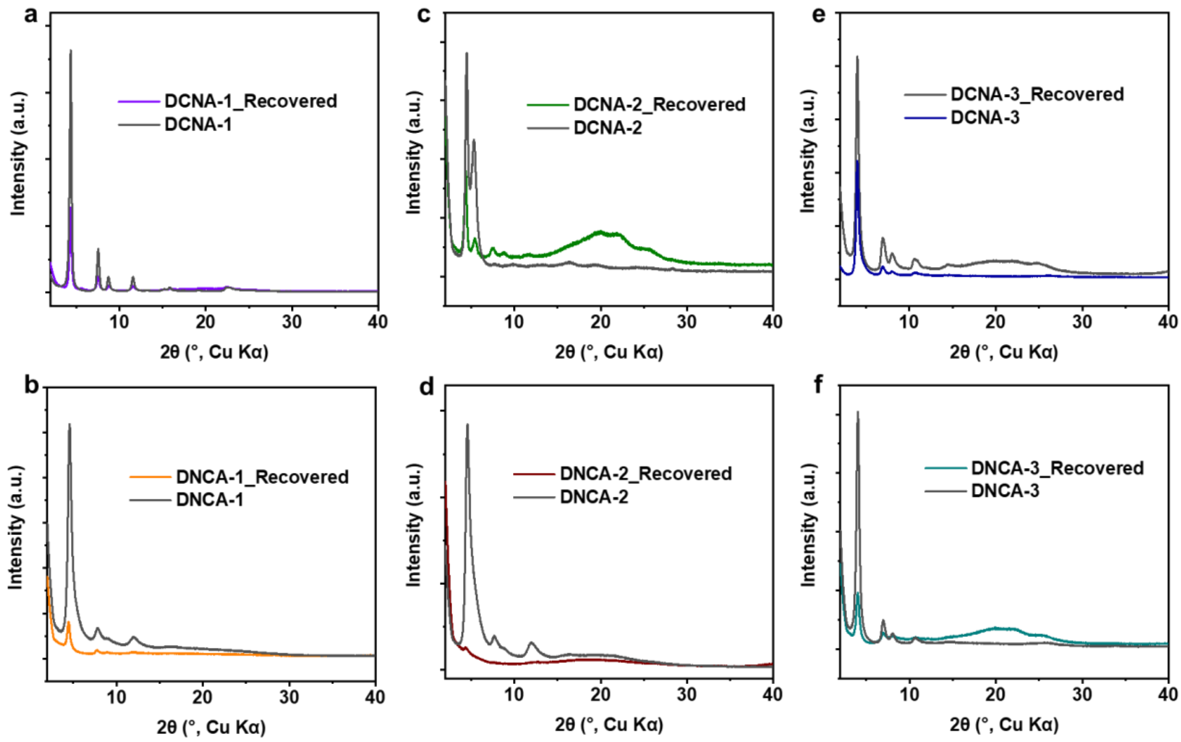

Supplementary Figure 23. PXRD patterns of the COFs recovered after photocatalysis in 0.1 M AC aqueous solution. (a) DCNA-1, (b) DNCA-1, (c) DCNA-2, (d) DNCA-2, (e) DCNA-3, (f) DNCA-3.

Supplementary Note 7. Quantum chemical calculations

The conduction band minimum (CBM) and valence band maximum (VBM) positions (relative to the vacuum level) were obtained from periodic DFT calculations. The HOMO and LUMO levels from one-pore cluster calculations were also calculated for comparison (Supplementary Table 5). In the case of periodic calculations for protonated COFs, only the single-H^+ case was considered, and the ascorbate counterion was included as shown in Supplementary Fig. 24. From the periodic HSE calculations, the CBM shifts (ΔCB) are -0.85 eV and -0.62 eV upon protonation, which is well consistent with the shifts of the experimental band edges (the corresponding experimental shift values are -0.64 and -0.40 eV, respectively). The cluster models also give the same trend.

A straightforward explanation for the decrease of the CB position can be that imine bond display a -M-effect when linked via the C-atom and a +M-effect when linked via the N-atom. Thus, in DNCA the “D” group becomes an even stronger electron donor, the “A” moiety an even stronger acceptor, which considering the MO model should yield in smaller band gaps and reduced CB potentials, in comparison to DCNA, where the effect is reversed. However, this effect cannot be easily deduced from the theoretical calculations.

Supplementary Table 5. Energy in eV of VBM and CBM from experiment and theory.

	Experiment		Periodic model, HSE		Cluster model, CAM-B3LYP	
	VBM	CBM	VBM	CBM	HOMO	LUMO
DCNA-1	-6.04	-3.56	-5.50	-2.88	-6.61	-1.33
DCNA-1+H^+	-6.10	-4.20	-5.88 (-4.76) [a]	-3.73	-7.28	-5.28
DCNA_1+6H^+					-14.21	-10.55
DNCA-1	-6.08	-4.02	-5.10	-3.14	-6.28	-3.24
DNCA-1+H^+	-5.98	-4.42	-5.36 (-4.87) [a]	-3.76	-6.94	-5.70
DNCA_1+6H^+					-14.01	-11.81

[a] Values in parentheses are taking the ascorbate counterion into account.

One-pore cluster model were also built up. In extreme cases, both one- (DCNA-1+H^+) or six-fold (DCNA-1+6H^+) protonation was considered for the protonated COFs. No counterions were included. HOMO-LUMO gaps and TD-DFT excitation energies for DCNA-1, DNCA-1, and their protonated forms are reported and compared to experimental excitation energies (Supplementary Table 6). While the HOMO-LUMO gaps are typically too large, the TD-DFT excitation energies fit better to qualitatively resemble the experimental trends. For “bright” TD-DFT transitions, oscillator strengths are also shown.

Supplementary Table 6. Excitation energies for DCNA-1, DNCA-1, DCNA-1_AC, and DNCA-1_AC from experiment and theory. The oscillator strengths f_{osc} are given for the first “bright” excitation.

	Experimental	Calculated CAM-B3LYP/ def2-SVP			
	UV-Vis DRS	HOMO-LUMO gap	1st excitation	1st excitation [a]	f_{osc}
DCNA-1	2.38 eV<br>(520 nm)	5.28 eV<br>(235 nm)	3.24 eV<br>(382 nm)	3.39 eV<br>(366 nm)	4.34
DCNA-1+H^+	1.81 eV<br>(685 nm)	2.16 eV<br>(575 nm)	1.75 eV<br>(708 nm)	2.42 eV<br>(513 nm)	1.32
DCNA-1+6H^+		3.66 eV<br>(339 nm)	2.34 eV<br>(529 nm)	2.44 eV<br>(508 nm)	2.54
DNCA-1	1.92 eV<br>(645 nm)	4.73 eV<br>(262 nm)	3.07 eV<br>(405 nm)	3.18 eV<br>(389 nm)	3.37
DNCA-1+H^+	1.35 eV<br>(916 nm)	1.59 eV<br>(780 nm)	1.20 eV<br>(1031 nm)	2.03 eV<br>(612 nm)	0.96
DNCA-1+6H^+		3.50 eV	2.10 eV	2.20 eV	2.14

		(354 nm)	(591 nm)	(565 nm)	

[a] Disregarding "dark" excitation

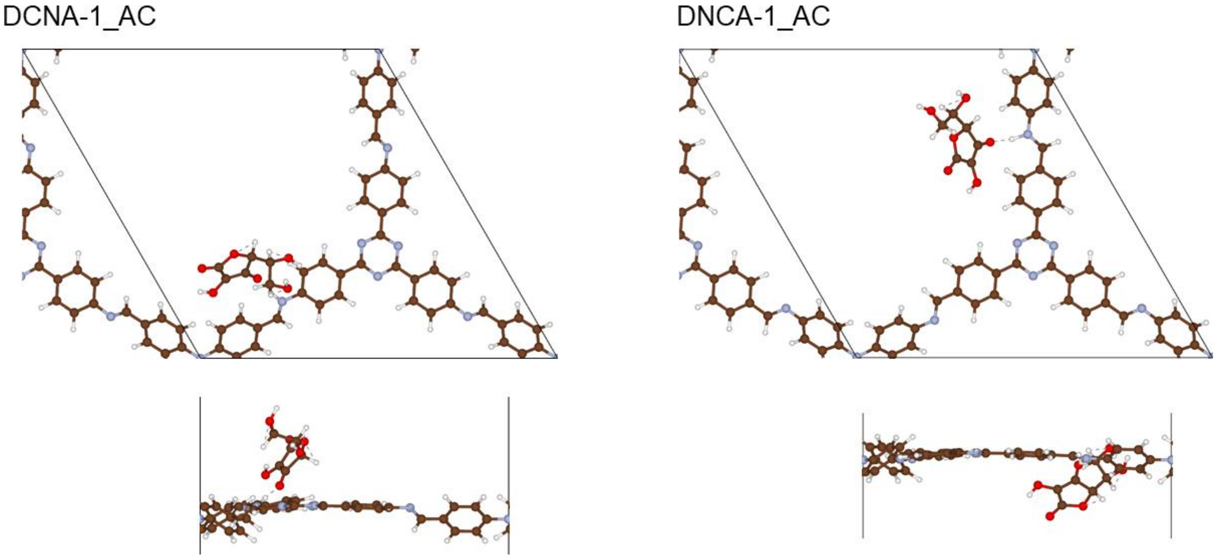

Supplementary Figure 24. Periodic structure models of DCNA-1_AC and DNCA-1_AC. The ascorbate anion was placed at the protonated imine group, creating a hydrogen bond. C, N, O, and H are shown in brown, blue, red, and white, respectively.

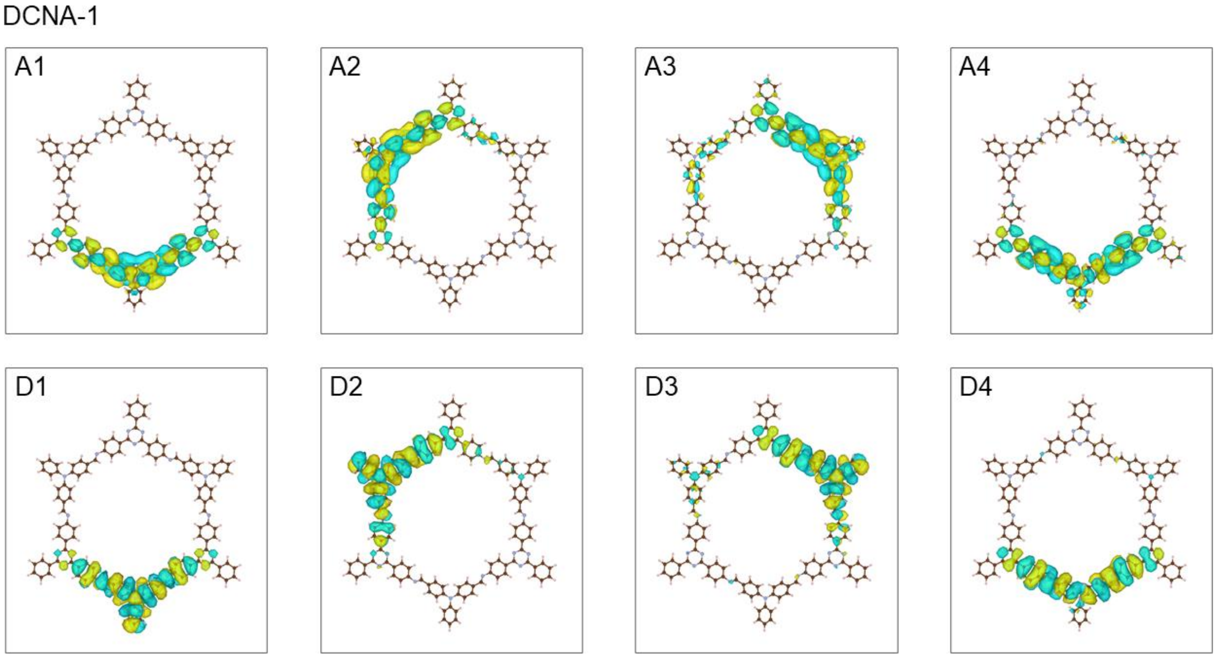

Supplementary Figure 25. Natural transition orbitals (NTOs) for the lowest dipole-allowed excitation of DCNA-1. Donor and acceptor orbitals are denoted D and A, respectively, and ordered by their weights, which are 0.53, 0.23, 0.10, and 0.07, respectively.

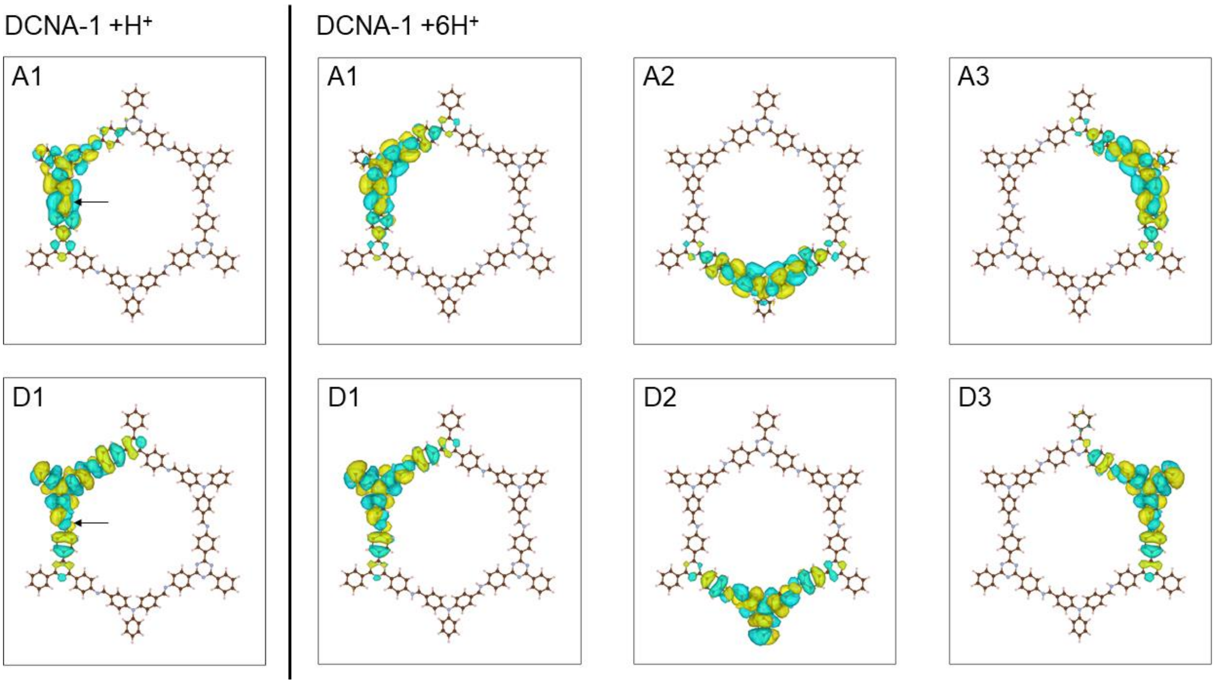

Supplementary Figure 26. Natural transition orbitals (NTOs) for the lowest dipole-allowed excitation of DCNA-1_AC. The arrow indicates the protonation site for the singly protonated cluster. Donor and acceptor orbitals are denoted D and A, respectively, and ordered by their weights, which are 0.96 (single protonation) and 0.61, 0.26, and 0.07, respectively

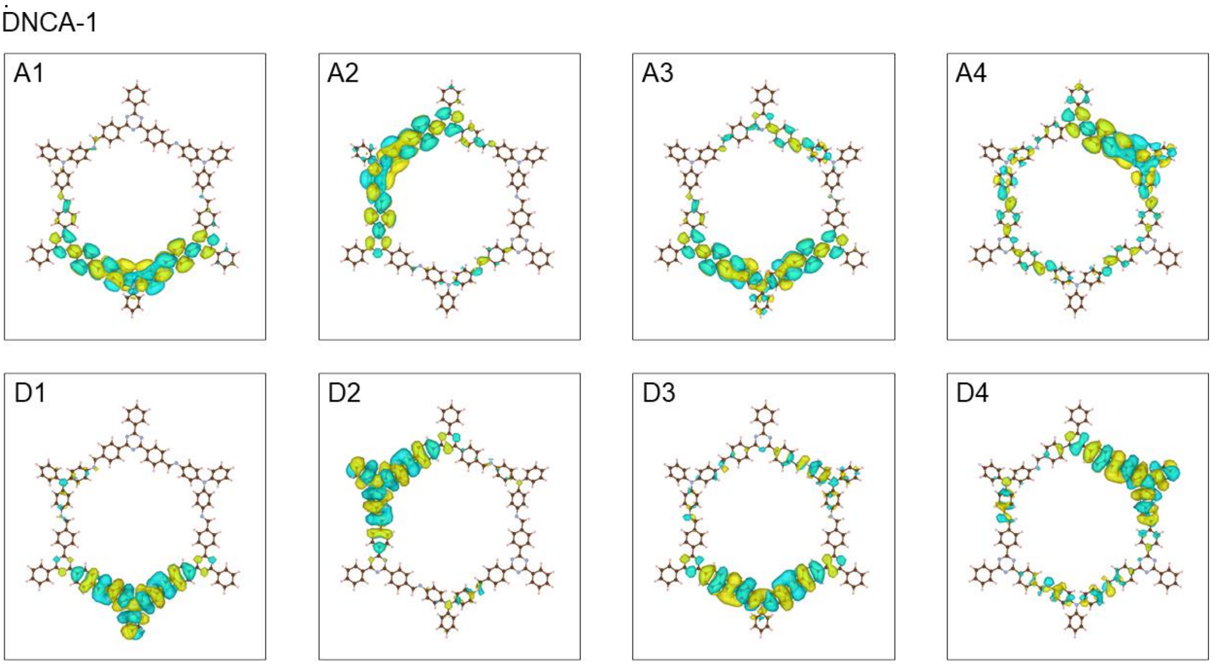

Supplementary Figure 27. Natural transition orbitals (NTOs) for the lowest dipole-allowed excitation of DNCA-1. Donor and acceptor orbitals are denoted D and A, respectively, and ordered by their weights, which are 0.52, 0.30, 0.06, and 0.05, respectively.

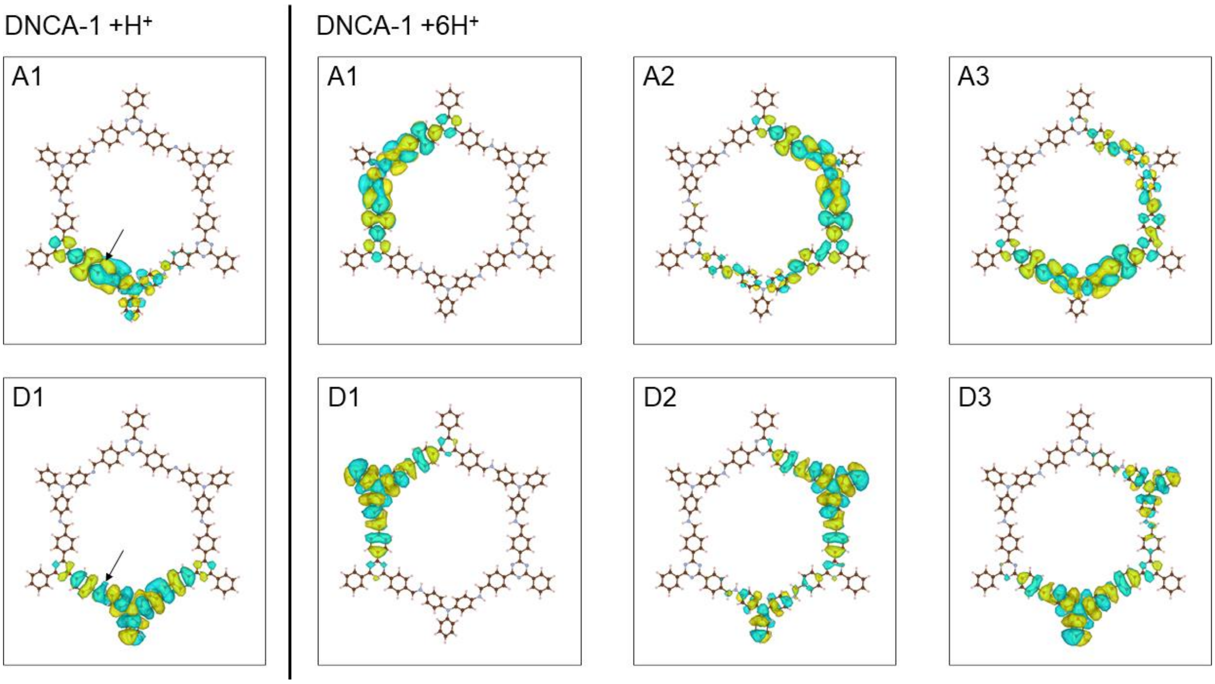

Supplementary Figure 28. Natural transition orbitals (NTOs) for the lowest dipole-allowed excitation of DNCA-1_AC. The arrows indicate the protonation site for the singly protonated cluster. Donor and acceptor orbitals are denoted D and A, respectively, and ordered by their weights, which are 0.98 (single protonation) and 0.63, 0.18, and 0.15, respectively.

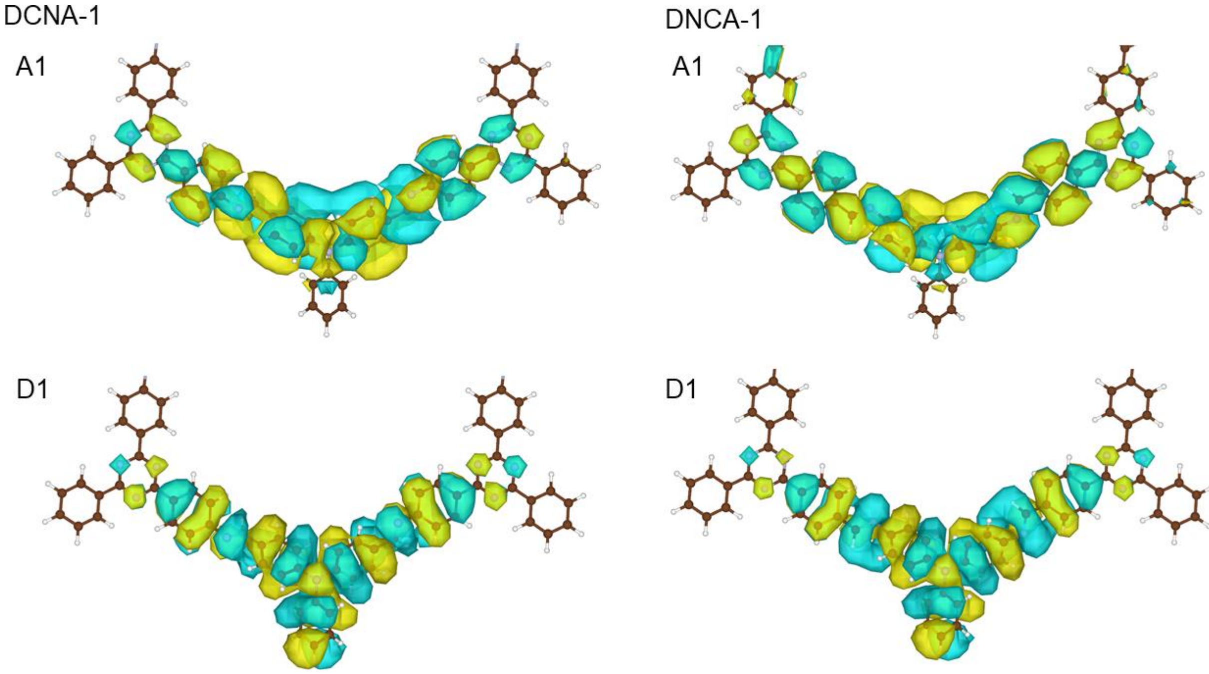

Supplementary Figure 29. A closer look at the natural transition orbitals (NTOs) for the lowest dipole-allowed excitation of DCNA-1 and DNCA-1.

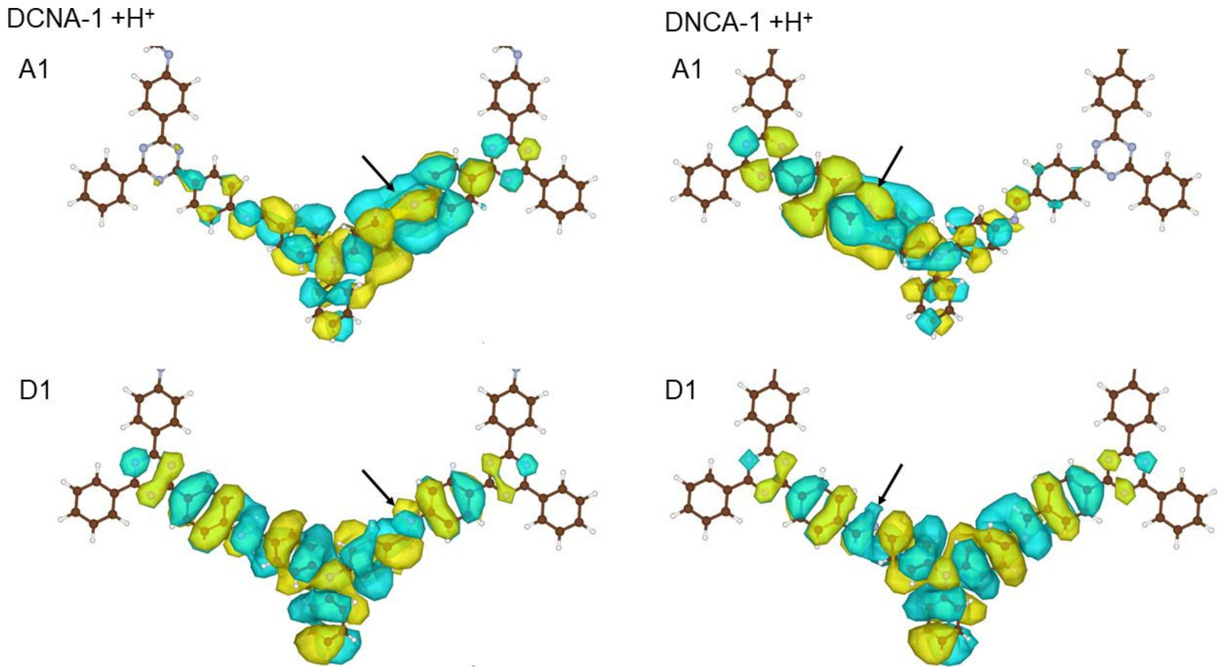

Supplementary Figure 30. A closer look at the natural transition orbitals (NTOs) for the lowest dipole-allowed excitation of DCNA-1_AC and DNCA-1_AC. The arrows indicate the protonation site.

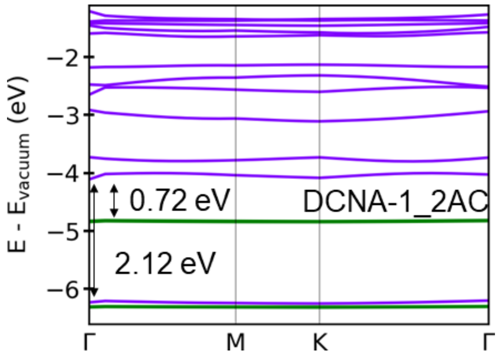

Supplementary Figure 31. Calculated HSE electronic band structures of the two-imine-sites-protonated DCNA-1 COFs. The decision of introducing two protonation sites is based on the XPS results (Supplementary Table 1).

Based on the XPS data (Supplementary Table 1), ~50% of the imine sites are protonated in DCNA-1_AC. Thus, the electronic band structure of the two-imine-sites-protonated (2/3 of all imine sites) DCNA-1 COFs are further simulated. The bandgap of the DCNA-1_2AC does not change much when adding one more proton and one more ascorbate anion (2.15 eV to 2.12 eV). The gap between ascorbate valence band and COF conduction band is reduced, but this shouldn't be important for the photophysics/photochemistry of the COF.

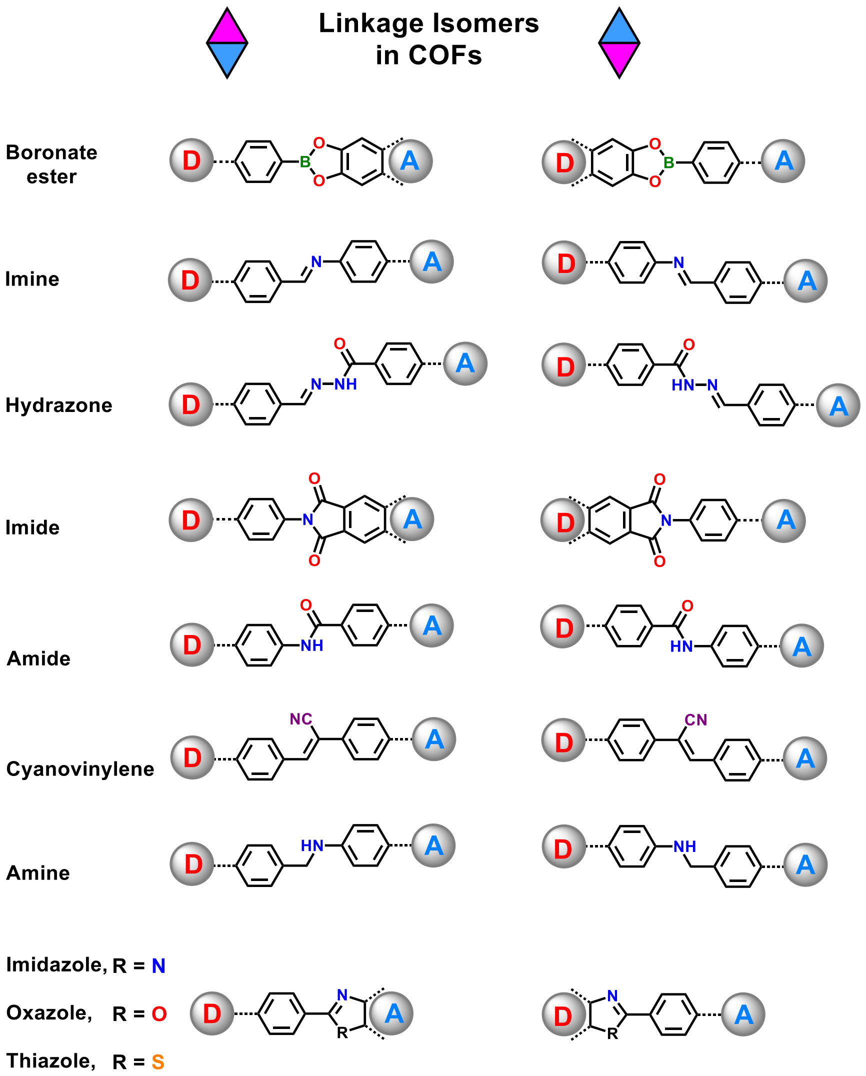

Supplementary Figure 32. The scope of the linkages with constitutional isomerism in COFs synthesis.

Reference

1. Kresse, G. & Furthmüller, J. Efficiency of ab-initio total energy calculations for metals and semiconductors using a plane-wave basis set. *Comput. Mater. Sci.* **6**, 15–50 (1996).
2. Kresse, G. & Furthmüller, J. Efficient iterative schemes for *ab initio* total-energy calculations using a plane-wave basis set. *Phys. Rev. B* **54**, 11169–11186 (1996).
3. Perdew, J. P., Burke, K. & Ernzerhof, M. Generalized Gradient Approximation Made Simple. *Phys. Rev. Lett.* **77**, 3865–3868 (1996).
4. Heyd, J., Scuseria, G. E. & Ernzerhof, M. Erratum: "Hybrid functionals based on a screened Coulomb potential" [J. Chem. Phys. 118, 8207 (2003)]. *J. Chem. Phys.* **124**, 219906 (2006).
5. Heyd, J., Scuseria, G. E. & Ernzerhof, M. Hybrid functionals based on a screened Coulomb potential. *J. Chem. Phys.* **118**, 8207–8215 (2003).
6. Grimme, S., Antony, J., Ehrlich, S. & Krieg, H. A consistent and accurate ab initio parametrization of density functional dispersion correction (DFT-D) for the 94 elements H-Pu. *J. Chem. Phys.* **132**, 154104 (2010).
7. Grimme, S., Ehrlich, S. & Goerigk, L. Effect of the damping function in dispersion corrected density functional theory. *J. Comput. Chem.* **32**, 1456–1465 (2011).
8. Neese, F. The ORCA program system. *WIREs Comput. Mol. Sci.* **2**, 73–78 (2012).
9. Krishnan, R., Binkley, J. S., Seeger, R. & Pople, J. A. Self-consistent molecular orbital methods. XX. A basis set for correlated wave functions. *J. Chem. Phys.* **72**, 650–654 (1980).
10. Stephens, P. J., Devlin, F. J., Chabalowski, C. F. & Frisch, M. J. Ab Initio Calculation of Vibrational Absorption and Circular Dichroism Spectra Using Density Functional Force Fields. *J. Phys. Chem.* **98**, 11623–11627 (1994).
11. Yanai, T., Tew, D. P. & Handy, N. C. A new hybrid exchange–correlation functional using the Coulomb-attenuating method (CAM-B3LYP). *Chem. Phys. Lett.* **393**, 51–57 (2004).
12. Becke, A. D. Density-functional thermochemistry. III. The role of exact exchange. *J. Chem. Phys.* **98**, 5648–5652 (1993).
13. Lee, C., Yang, W. & Parr, R. G. Development of the Colle-Salvetti correlation-energy formula into a functional of the electron density. *Phys. Rev. B* **37**, 785–789 (1988).
14. Weigend, F. & Ahlrichs, R. Balanced basis sets of split valence, triple zeta valence and quadruple zeta valence quality for H to Rn: Design and assessment of accuracy. *Phys. Chem. Chem. Phys.* **7**, 3297 (2005).
15. Runge, E. & Gross, E. K. U. Density-Functional Theory for Time-Dependent Systems. *Phys. Rev. Lett.* **52**, 997–1000 (1984).
16. Martin, R. L. Natural transition orbitals. *J. Chem. Phys.* **118**, 4775–4777 (2003).
17. Yang, J. *et al.* Protonated Imine-Linked Covalent Organic Frameworks for Photocatalytic Hydrogen Evolution. *Angew. Chemie - Int. Ed.* **60**, 19797–19803 (2021).
18. El-Mahdy, A. F. M. *et al.* Strategic design of triphenylamine- and triphenyltriazine-based two-dimensional covalent organic frameworks for CO2 uptake and energy storage. *J. Mater. Chem. A* **6**, 19532–19541 (2018).
19. Haase, F. *et al.* Tuning the stacking behaviour of a 2D covalent organic framework through non-covalent interactions. *Mater. Chem. Front.* **1**, 1354–1361 (2017).
20. Keller, N. *et al.* Enforcing Extended Porphyrin J-Aggregate Stacking in Covalent Organic Frameworks. *J. Am. Chem. Soc.* **140**, 16544–16552 (2018).
21. Yan, J., Zhang, X., Zheng, W. & Lee, L. Y. S. Interface Engineering of a 2D-C 3 N 4 /NiFe-LDH Heterostructure for Highly Efficient Photocatalytic Hydrogen Evolution. *ACS Appl. Mater. Interfaces* **13**, 24723–24733 (2021).
